# Complex protein interactions mediate *Drosophila* Lar function in muscle tissue

**DOI:** 10.1371/journal.pone.0269037

**Published:** 2022-05-27

**Authors:** Jessica Kawakami, David Brooks, Rana Zalmai, Steven D. Hartson, Samuel Bouyain, Erika R. Geisbrecht

**Affiliations:** 1 Department of Cell and Molecular Biology and Biochemistry, University of Missouri-Kansas City, Kansas City, MO, United States of America; 2 Department of Biochemistry and Molecular Biophysics, Kansas State University, Manhattan, Kansas, United States of America; 3 Department of Biochemistry and Molecular Biology, Oklahoma State University, Stillwater, OK, United States of America; UNIVERSITY OF CENTRAL FLORIDA, UNITED STATES

## Abstract

The type IIa family of receptor protein tyrosine phosphatases (RPTPs), including Lar, RPTPσ and RPTPδ, are well-studied in coordinating actin cytoskeletal rearrangements during axon guidance and synaptogenesis. To determine whether this regulation is conserved in other tissues, interdisciplinary approaches were utilized to study Lar-RPTPs in the *Drosophila* musculature. Here we find that the single fly ortholog, *Drosophila* Lar (Dlar), is localized to the muscle costamere and that a decrease in Dlar causes aberrant sarcomeric patterning, deficits in larval locomotion, and integrin mislocalization. Sequence analysis uncovered an evolutionarily conserved Lys-Gly-Asp (KGD) signature in the extracellular region of Dlar. Since this tripeptide sequence is similar to the integrin-binding Arg-Gly-Asp (RGD) motif, we tested the hypothesis that Dlar directly interacts with integrin proteins. However, structural analyses of the fibronectin type III domains of Dlar and two vertebrate orthologs that include this conserved motif indicate that this KGD tripeptide is not accessible and thus unlikely to mediate physical interactions with integrins. These results, together with the proteomics identification of basement membrane (BM) proteins as potential ligands for type IIa RPTPs, suggest a complex network of protein interactions in the extracellular space that may mediate Lar function and/or signaling in muscle tissue.

## Introduction

The reversible phosphorylation of tyrosine is a post-translational modification utilized for signal transduction and mediated by the opposing actions of protein tyrosine kinases and protein tyrosine phosphatases [1]. Tyrosine phosphorylation is particularly important to metazoans, as it plays a central role in growth and development [2]. Accordingly, aberrant phosphorylation is associated with human diseases, such as cancer and diabetes [3, 4]. An understudied class of proteins in phosphotyrosine signaling, the receptor protein tyrosine phosphatases (RPTPs), are a family of single-pass transmembrane proteins whose architecture include an extracellular domain resembling those of cell adhesion molecules (CAMs) and intracellular single or tandem tyrosine phosphatase domains [5]. These receptors mediate cell-cell or cell-matrix adhesion, yet the exact identities of the RPTP extracellular ligands are predominantly unknown and the physiological roles played by these interactions, beyond those described in neural tissues [6], are also ill-defined.

**Fig 1:**
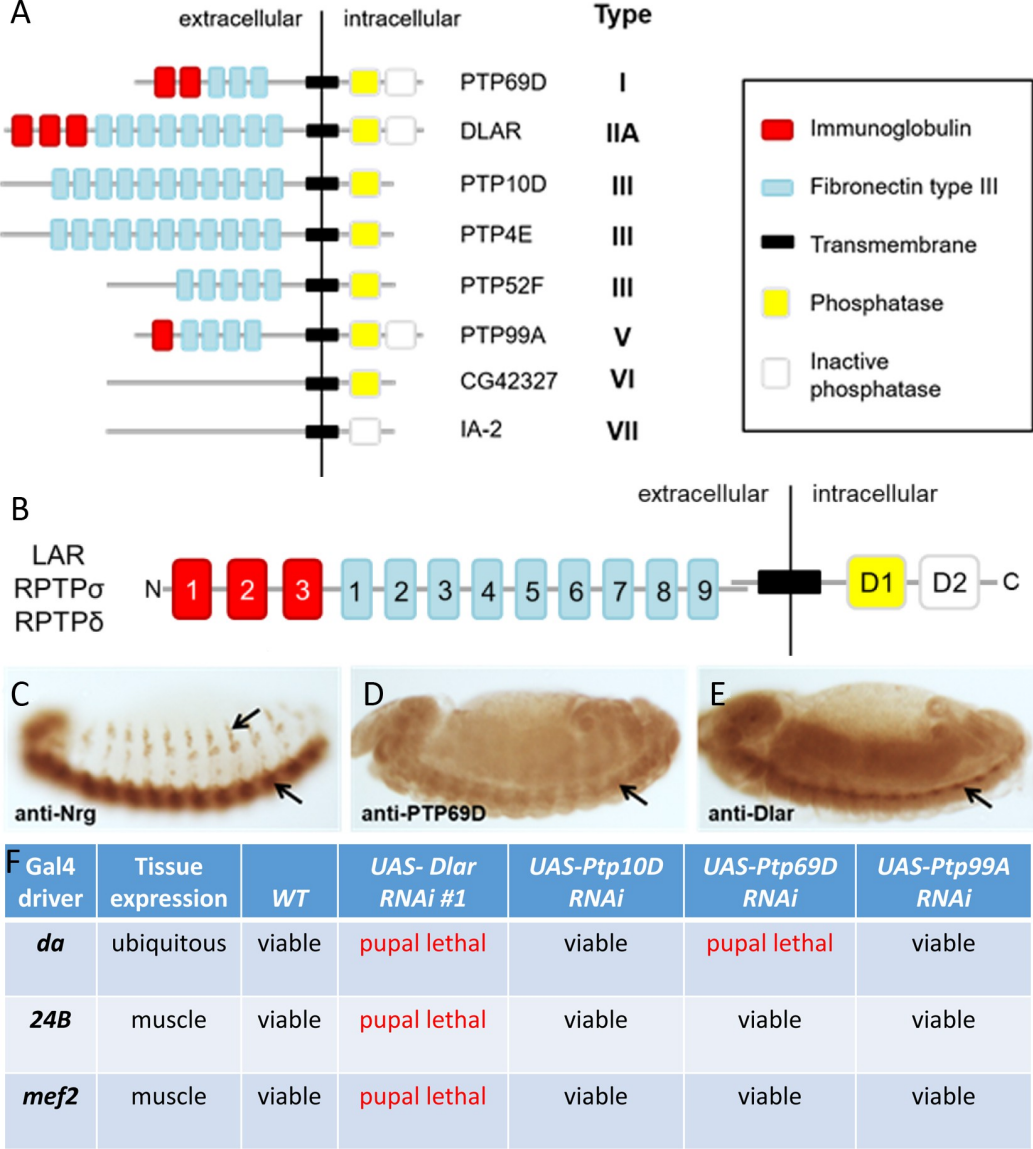
Dlar is required in muscle tissue. (A) There are eight RPTPs in the Drosophila genome. Type (I-VII) is determined by the orthology relationship to human RPTPs. Key domains are described in the legend (right). (B) Schematic representation of the Lar-RPTP domains. (C-E) Immunohistochemical stainings of WT stage 13 embryos. (C) Neuroglian (Nrg) is expressed exclusively in the ventral nerve cord (VNC, lower arrow) and peripheral neurons (upper arrow). While PTP69D (D) and Dlar (E) are also present in the VNC (arrow), mesoderm expression is also prevalent. (F) Viability results of GAL4/UAS knockdown experiments using RNAi against the indicated RPTPs at 29°C. A cross was considered viable by the presence of adults eclosing after pupation.

The human genome contains twenty-one genes encoding RPTPs–compared to eight in the *Drosophila* genome–that are classified into eight subtypes based upon amino acid sequence alignments of the intracellular phosphatase domains (**Fig 1A**) [7, 8]. Although several RPTPs remain orphan receptors, the known ligands encompass a range of proteins including: extracellular matrix (ECM) proteins [9], CAMs [10], and growth factors [11]. In vertebrates, this diversity is typified by the leukocyte common antigen related (Lar) and its two homologs RPTPδ/PTPRD and RPTPσ/PTPRS, which form the type IIa family of RPTPs. These Lar-RPTPs are arguably the best characterized subtype of RPTPs and share ~ 90% amino acid sequence identity in their phosphatase domains and ~75% sequence identity with the single invertebrate ortholog, *Drosophila* Lar (Dlar) [12, 13]. The ectodomains of Lar-RPTPs resemble immunoglobulin (Ig)-superfamily CAMs and include three Ig-like domains followed by four to nine fibronectin type III (FN) repeats (**Fig 1B**) [14]. Typically, the intracellular membrane proximal domain (D1) is catalytically active and the membrane distal (D2) is a pseudophosphatase that plays a regulatory role [15]. For example, recent work indicates that aggregation of type IIa RPTPs through interactions mediated by the pseudophosphatase domain decreases receptor phosphatase activity [16].

RPTPs were initially considered neural-specific receptors after the characterization of expression patterns across species–namely Dlar, PTP99A, PTP10D and PTP69D in *Drosophila* [17–19], chicken RPTPσ [20], and human RPTPδ [14]. Due to the redundancy of RPTP function in mammals, key insights into neural regulation were confirmed in *Drosophila*, whereby *Dlar* [21], *Ptp69D* [22] or *Ptp52F* [23] single mutants display motor axon pathfinding defects during innervation of body wall muscle targets. In contrast, mutants of *Ptp99A* and *Ptp10D* require concomitant mutations in *Dlar*, *Ptp69D*, or *Ptp52F* to produce neural phenotypes [22–26]. Double, triple or quadruple mutants reveal intricate interactions that can be synergistic or antagonistic [27].

Despite enrichment of the mammalian type IIa RPTPs in neuronal tissues, the overlapping expression patterns obfuscate their function in motor axon guidance. While RPTPσ and RPTPδ single mutant mice are viable, RPTPσ/δ double mutant mice die at birth from respiratory failure [28]. This is attributed to defective late-stage extension of motor neurons, which causes abnormally thin skeletal muscle in the diaphragm and limbs. Interestingly, Northern blot analyses have revealed that genes encoding human Lar, RPTPσ, and RPTPδ are expressed in heart and muscle tissues as well as the brain. Similarly, RNA *in situ* hybridization in rodents localize transcripts of RPTPσ and RPTPδ to tissues of neuronal and mesodermal origin, including the striated musculature [14, 29]. Although these lines of evidence suggest that type IIa RPTPs might function beyond neuronal tissues, characterization in other tissues in both vertebrates and invertebrates has been sparse due to early lethality caused by defective neural development [21].

*Drosophila* provides an exemplary model to address the potential functions of Lar and related proteins outside of the nervous system without the functional redundancy present in vertebrate models. This work highlights a previously unknown role for Dlar in the *Drosophila* musculature. RNAi-mediated knockdown of the single Dlar ortholog in muscle is pupal lethal and alters the organization of sarcomeric F-actin and costamere-associated integrins. Despite identification of a conserved KGD integrin binding motif in the FN domains of Dlar and its conserved mammalian family members, analysis of this KGD motif by X-ray crystallography shows this region is not normally accessible for integrin binding. Overall, this work suggests that the function of Lar in the musculature likely requires additional, yet unknown, protein and signaling interactions.

## Materials and methods

### Drosophila genetics

The following stocks were obtained from the Bloomington *Drosophila* Stock Center (BL) or the Vienna *Drosophila* Stock Center (VDRC): wild-type (*WT*) strain refers to *w*^*1118*^ (BL3605); *24B*-Gal4 (BL1796), *mef2*-Gal4 (BL27390); *da-*Gal4 (BL55850); UAS-*Dlar RNAi #1* (TRiP.HMS00822; BL34965); UAS-*Dlar RNAi #2* (TRiP.HMS02186; BL40938); UAS-*Dlar RNAi #3* (TRiP.GL01589; BL43979); UAS-*Ptp10D RNAi* (TRiP.HMS01917; BL39001); UAS-*Ptp69D RNAi* (TRiP.JF03399; BL29462); UAS-*Ptp99A RNAi* (TRiP.JF01858; BL25840), and UAS-*Glutactin* (*Glt) RNAi* (KK110236; v101918). Stocks and fly crosses were raised at 25°C on standard cornmeal medium unless otherwise indicated.

### qPCR analysis

Knockdown of Dlar was verified by quantitative real-time PCR (qPCR). Crosses were set with the *da*-Gal4 driver and reared at 29°C to maximize expression of RNAi [30]. Between 1 and 5 larvae were homogenized for each biological replicate and total RNA was extracted and purified using the RNeasy Mini Kit (Qiagen). 150 ng– 600 ng of RNA was used to make single stranded cDNA using the SuperScript III First-Strand Synthesis System Kit (Invitrogen). The cDNA solution was diluted 1:10, 1:25 or 1:50 and mixed with iQ SYBR Green Supermix (Bio-Rad). Primers for the qPCR reactions were synthesized by Integrated DNA Technologies (IDT, Coralville, IA): *Dlar* forward primer 5’-CCCAGATGGTCGACAATAGCG-3’; *Dlar* reverse primer 5’-CCGGCTCCCATCGATGTGTA-3’; *Glt* forward primer 5’- AGC CTC ACT AGC CAC CAA C; *Glt* reverse primer 5’- CTT CCA GAG GCG GTG CTC; *rp49* forward primer 5’-GCCCAAGGGTATCGACAACA-3’; *rp49* reverse primer 5’-GCGCTTGTTCGATCCGTAAC-3’. qPCR assays were carried out on the CFX96 Touch Real-Time PCR Detection System with CFX Manager Software (Bio-Rad). Three technical replicates for each biological sample were averaged to generate Ct values. The differential normalized fold change was performed using the ΔΔCt method.

### Immunohistochemistry and imaging

#### Embryo staining

Embryos were collected on apple juice agar plates and aged at 25°C to obtain stage 12–18 embryos. Embryos were washed from the plates with 0.7%NaCl/0.04%Triton X-100, transferred to a collection basket, and placed in freshly prepared 50% bleach for 3–4 min until the chorion could no longer be visualized. A paintbrush was used to transfer embryos to a glass vial containing a 1:1 mixture of heptane:4% formaldehyde (Polysciences) in PEM buffer (0.1 M Pipes, pH 8.0; 2 mM MgSO4; 1 mM EGTA) and subjected to vigorous shaking for 20 min at room temperature. The bottom layer of fixative was removed and replaced by an equal volume of methanol (Thermo Fisher Scientific). Vigorous shaking of the vial for ~1 min caused removal of the vitelline membrane and embryo sedimentation. The resulting embryos were transferred to a microcentrifuge tube, washed three times with PBT, and blocked with 5% normal goal serum (NGS) in PBT for 30 min. Mouse primary antibodies were incubated with embryos overnight at 4°C, washed three times with PBT, incubated with secondary biotinylated antibodies for 2 hr at room temperature and washed again three times with PBT. The following primary antibodies were purchased from the Developmental Studies Hybridoma Bank (DSHB): mouse anti-Neuroglian (Nrg) BP104 (1:10), mouse anti-PTP69D 3F11 (1:5), or mouse anti-Dlar 9D82B3 (1:10). Colorimetric detection was performed using biotinylated anti-mouse IgG (1:200) and the Vectastain ABC Elite Kit (Vector Laboratories) according to the manufacturer’s instructions. *Larval muscle*. Wandering L3 larvae were collected from the side of a food vial, filleted and fixed in 4% formaldehyde as previously described [31]. Anti-Dlar FN5 (1:10) is described below and anti-integrin βPS CF.6G11 (1:50) was obtained from DSHB. Anti-mouse Alexa Fluor® 488 was used to visualize the primary antibodies [1:400 (Thermo Fisher Scientific)] and phalloidin 594 and DAPI 405 were used to label F-actin or nuclei, respectively. Tyramide signal amplification was utilized to amplify the Dlar signal (Vector Labs, Burlingame, CA). Confocal images were collected on an Olympus Fluoview 300 (UMKC) or a Zeiss 700 (KSU) and processed in ImageJ.

### Phenotypic quantitation and analysis

For quantitation of sarcomeric actin defects, crosses were reared at 25°C. L3 larval fillets were fixed and stained with phalloidin. Larvae were scored as percent defective based on a total of 80 muscles per larvae omitting hemisegments near the anterior and posterior, as well as dorsal muscles to circumvent quantitation of artifacts from dissection. Percent defective for each cross was analyzed for 20 larvae in GraphPad Prism 6.0 by comparing the mean of each group to *WT* using the Mann-Whitney U test. Larval locomotion was analyzed as previously described [32].

### Protein expression and purification

Dlar FN5 (residues 706–812) was amplified by PCR from a *Drosophila* cDNA library and cloned into a pT7HMP vector. Protein expression in *E*. *coli* BL21(DE3) cells resulted in a hexahistidine fusion protein with a human rhinovirus 3C protease cleavage site. Following homogenization and lysis of cells, protein was purified by batch binding on Ni-NTA resin (Thermo Fisher Scientific). After proteolytic cleavage of the hexahistidine tag, protein was purified by immobilized-metal affinity and ion exchange chromatography. Dlar FN4-FN6 (residues 611–907) was purified as above, except the construct was obtained from IDT as a de novo synthesized gene fragment. Dlar Ig1-Ig2 (residues 32–237) was expressed in a modified pET32 vector (MilliporeSigma), called pET32HP, expressing a thioredoxin tag, a hexahistidine tag and a human rhinovirus 3C protease cleavage site [33] and purified as previously described [34]. The remaining constructs were cloned into pET32HP and purified as above; Dlar FN4-FN5 (residues 608–812) was cloned by PCR and mouse Lar FN5 (residues 710–810) was purchased from Genscript as a de novo synthesized gene fragment. Transient expression of proteins in HEK293 cells from a pHLSEC2Fc vector results in a fusion protein of Human IgG Fc. Conditioned media was dialyzed against 20 mM Tris pH 7.5, 150 mM NaCl and affinity purified on protein A-agarose [33]. The following proteins were transiently expressed in HEK293 cells; sDlar, human RPTPδ FN4-FN6, mouse RPTPδ FN4-FN7, mouse Lar FN4-FN7, mouse CNTN1 FN1-FN3, and mouse CNTN6 FN1-FN3. Western blotting with rabbit anti-human IgG Fc confirmed expression of fusion proteins.

### Generation of Dlar antibodies

Custom Dlar antibodies were raised in rabbits against the extracellular Dlar domain fragments Ig1-Ig2 or FN5. These purified proteins were sent to Pocono Rabbit Farm and Laboratory and antibodies were generated using their 70-day protocol. Antibodies from the exsanguination bleed were purified on an affinity chromatography column created by covalent attachment of antigen to NHS-activated agarose (Pierce). Western blotting was used to verify the anti-Dlar FN5 used for tissue staining. L3 larvae of the indicated genotypes (*mef2>lacZ*, *mef2>Dlar OE*, *da>lacZ*, or *da>Dlar RNAi*) were placed into SDS sample buffer, boiled at 95°C for 3 min, homogenized, boiled for an additional 10 min at 95°C, and centrifuged at 20,000xg for 1 min to pellet debris. The resulting protein samples were separated by sodium dodecyl sulfate polyacrylamide gel electrophoresis (SDS-PAGE), transferred to polyvinyl difluoride (PVDF) membranes (Pierce Biotechnology, Inc., Waltham, MA), and probed with rabbit anti-Dlar FN5 (1:1000) and mouse anti-ATP5α (1:20000–1:140000, Abcam, Cambridge, United Kingdom) as a loading control. Horseradish Peroxidase (HRP) conjugated secondary antibodies (1:5000–1:10000, GE Healthcare, Chicago, IL) were developed using OneStep Ultra TMB (Thermo Fisher Scientific) or Pierce ECL Plus (Thermo Fisher Scientific) and imaged with the FluorChem M system (Protein Simple, San Jose, CA) or the LI-COR Odyssey XF (LI-COR Biosciences, Lincoln, NE) as indicated. Quantification of Western blot protein levels was performed in ImageJ.

### X-ray crystallography

Crystals were grown by hanging drop diffusion at 20°C. Conditions used for crystallization and cryoprotection are listed in **Table 1**. X-ray diffraction data was collected at beamlines 22-BM and 22-ID of the Advanced Photon source at Argonne National Laboratory. Diffraction data was processed with HKL2000 [35] or with MOSFLM/Aimless integrated in CCP4 [36]. The structure of the FN5 domain of Dlar was initially solved using anomalous signal from two bound Zn^2+^ ions. Although less than 10 residues were built initially by the AutoSol routine in Phenix [37], the electron density maps were readily interpretable and about 2/3 of the domain could be built manually using Coot [38]. A good model for FN5 was then obtained by using a partially built model as a molecular replacement model and using the Phaser and AutoBuild routines implemented in Phenix. The final model for Dlar FN5 was then used to obtain initial models for mouse Lar FN5 and human RPTPδ FN4-FN6 by molecular replacement. All structures were refined using Phenix. Figures were prepared using Chimera X [39].

**Table 1:** Crystallization and cryoprotection conditions.

Protein	Crystallization conditions	Cryoprotection conditions
Dlar FN5	100 mM HEPES pH 7.0, 200 mM Ammonium acetate, 3mM Zinc acetate, 25% (w/v) PEG 3350	100 mM HEPES pH 7.0, 200 mM Ammonium acetate, 5mM Zinc acetate, 25% (w/v) PEG 3350, 10% (w/v) PEG 400
Mouse Lar FN5	100mM Na-cacodylate pH 6.5, 1.4 M Na-citrate tribasic dehydrate	100mM Na-cacodylate pH 6.5, 1.4 M Na-citrate tribasic dihydrate, 30% (w/v) sorbitol
Human RPTPδ FN4-FN6	200 mM Magnesium formate, 20% (w/v) PEG 3350,	200 mM Magnesium formate, 20% (w/v) PEG 3350, 15% (v/v) glycerol

### Immunoprecipitation and affinity isolation from larval lysates

*WT* L3 larvae were harvested from food bottles and homogenized in the indicated lysis buffers (**S1 Table**). After centrifugation, the supernatant was pre-cleared with Rat IgG attached to NHS-Activated Sepharose or empty NHS-Sepharose. After pre-clearing, the supernatant was incubated with the following baits attached to NHS-Activated Sepharose: purified rabbit anti-Dlar Ig1-Ig2 antibodies, Dlar FN4-FN5 from over-expression in *E*. *coli*, or sDlar from HEK293 cells. Coupling efficiency was assessed by SDS-PAGE followed by Coomassie staining. Negative controls included rabbit IgG or mouse CNTN4 FN1-3 attached to NHS-Activated Sepharose. After incubation, Sepharose was harvested by low speed centrifugation and washed three times with lysis buffer minus detergent. After the final spin, Sepharose was drained dry with a gel loading tip and snap frozen in liquid nitrogen for shipment to the Recombinant DNA/Protein Resource Facility at Oklahoma State University for analysis by LC–MS/MS.

### Affinity isolation from cell culture

Mammalian cell lines were grown to confluency in 150 cm^2^ plates at standard conditions. A total of five plates were harvested for each of the following cell lines: B35 (rat neuroblastoma), C2C12 (mouse myoblast), C6 (rat glioblastoma), HEK293 (human embryonic kidney), and Neuro2a (mouse neuroblastoma). Cell culture serum was aspirated from plates, cells were washed with Hanks Balanced Salt Solution with calcium and magnesium (HBSS) before gently scraping cells off of the bottom of plates and transferring to 15 ml conical tubes. Cells were centrifuged on low speed for 5 minutes and washed an additional two times with HBSS. After final spin, cells were resuspended and lysed in the indicated buffers (**S2 Table**). Lysates were pre-cleared with quenched CNBr-Activated Sepharose (Cytiva Lifesciences) and then lysate from each cell line was divided into four equal volumes and incubated with the following purified proteins attached to CNBr-Activated Sepharose: the FN4-FN7 domains of mouse Lar (MLar FN4-FN7) or mouse PTPRD (MPTPRD FN4-7) for bait and the FN1-FN3 domains of mouse CNTN1 (MCNTN1 FN1-3) or mouse CNTN6 (MCNTN6 FN1-3) served as negative controls. Sepharose was collected by centrifugation and washed three times with lysis buffer minus detergent before draining dry with a gel loading tip and snap frozen in liquid nitrogen as above.

### LC-MS/MS

Adsorption reactions were eluted with SDS-PAGE sample buffer and separated on 12% gels. Gels were fractionated and trypsinolyzed as described [40]. For solution digests, resins were eluted with 8M buffered urea, and then the eluates were reduced and alkylated, diluted to 2M urea, and digested with trypsin using standard methods. Peptides were purified from the solution digests using solid phase extraction on monolithic C18 pipet tips (Pierce). Peptides were analyzed on 75 micrometer x 40 cm nano-columns fabricated in house and packed with 3-micron beads of Magic AQ C18 resin (Michrom). Peptides were injected using a vented trap column configuration, and separated on 6%-35% linear acetonitrile gradients developed over a 100-minute chromatography run at 250 nL/min. Columns terminated in a stainless-steel emitter for peptide ionization within a Nanospray Flex ion ion source (Thermo). Peptide ions were analyzed in a quadrupole-Orbitrap “Fusion” mass spectrometer (Thermo) using a 3-second “top speed” data-dependent MS/MS scan method. In this method, peptide precursors were measured within the Orbitrap sector at a nominal resolution of 120,000. Ions were selected for MS/MS using the quadrupole, followed by CID fragmentation and analysis of the fragments using the ion trap detector. Peptides from each individual adsorption sample were subjected to three individual LC-MS/MS analyses, or technical replicates. Biological replicates of Dlar experiments included anti-Dlar and anti-Dlar Ig1-Ig2 experiments, and sDlar, Dlar Ig1-Ig2, Dlar FN4-FN5, and Dlar FN4-FN6 affinity isolations (**S1 Table**). Biological replicates for mammalian orthologs of Dlar included experiments from six mammalian cell lines (**S2 Table**). *Peptide identification and analysis*. Peptides were identified and quantified by using MaxQuant v1.5.3.8 [41] to search the RAW instrument files against databases of *Drosophila melanogaster*, rat, mouse, human sequences downloaded from Uniprot. Database searches utilized the default MaxQuant parameters. Statistical analysis and the generation of volcano plots was carried out in Perseus 1.2.0.16 [42]. Both normal intensities and label-free quantification (LFQ) intensities [43] were analyzed for the *Drosophila* larval experiments because biological replicates were temporally separated and there may have been differences in handling. Statistical threshold was determined on normalized data by T-tests (Benjamini-Hochberg FDR p < 0.05). Replicates were averaged and log_2_-transformed. Protein hits that were enriched at least 10-fold compared to control were considered statistically significance. Analysis of Lar and PTPRD were processed as above except only LFQ intensities were analyzed.

## Results and discussion

### Dlar is expressed in muscle tissue

We reassessed the embryonic protein expression patterns for select *Drosophila* RPTPs using anti-Dlar or anti-PTP69D monoclonal antibodies and compared the results to the neural-specific expression of Neuroglian (Nrg). Nrg is exclusively present in the developing peripheral nervous system (upper arrow) and ventral nerve cord (lower arrow) (**Fig 1C**). In contrast, PTP69D (**Fig 1D**) and Dlar (**Fig 1E**) proteins are present in the VNC (lower arrows) as well as mesodermal tissue. These data argue that PTP69D and Dlar may play a role in the developing musculature. To determine if *Drosophila* RPTPs functionally exhibit non-neuronal roles, we utilized the UAS/GAL4 system to target muscle tissue using RNA interference (RNAi) (**Fig 1F**) [44]. Mutations in *Dlar* and *Ptp69D* are reportedly lethal, whereas *Ptp10D* and *Ptp99A* mutants are viable and do not show an obvious neural phenotype [21, 22, 24]. Therefore, use of the ubiquitous *da-GAL4* driver served as a control to confirm the lethality of the *UAS-Dlar RNAi* and *UAS-Ptp69D RNAi*. Notably, induction of *Dlar RNAi #1* under control of the muscle drivers, *24B-GAL4* or *mef2-GAL4*, resulted in pupal lethality. However, *RNAi* knockdown of *Ptp10D*, *Ptp69D*, and *Ptp99A* in developing muscle tissue produced viable adults. Thus, the observed lethality was specific to Dlar ablation and not due to a general loss of the RPTPs.

**Fig 2:**
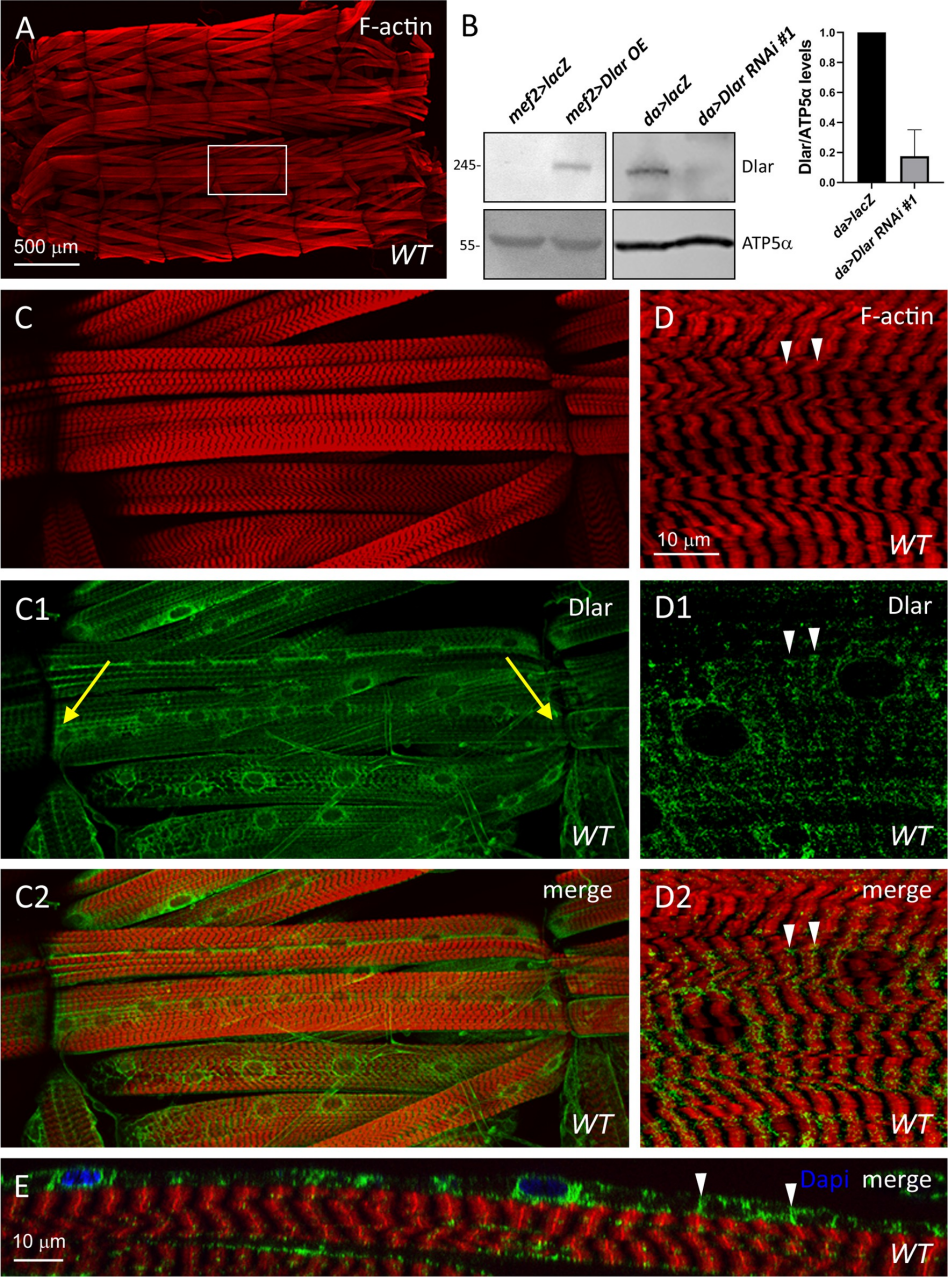
Dlar is localized to the larval muscle membrane at Z-discs. (A,C-E) WT L3 larval body wall muscles stained with phalloidin to label F-actin (red), Dlar (green) or DAPI (blue). (A) Low magnification image of a muscle fillet shows the repeated pattern of body wall muscles across hemisegments. White box indicates ventral longitudinal muscles depicted in C-C2. (B) Western blots of larval lysates using affinity purified rabbit anti-Dlar FN5 or anti-ATP5α (loading control). Dlar overexpression (Dlar OE) in muscle tissue using mef2-Gal4 obscures the signal of endogenous Dlar in control mef2>lacZ larvae (left panel). Ubiquitous RNAi knockdown by da-Gal4 results in a decrease in Dlar protein levels (right panel). Bar graph shows the relative levels of Dlar/ATP5α in control and Dlar RNAi larvae. Mean +/- SD. N = 3 biological replicates for each genotype. (C-C2, D-D2) Representative low (C-C2) or high (D-D2) magnification images of ventral longitudinal muscles indicating that Dlar localization partially overlaps with Z-discs in the center of F-actin striations (D-D2, white arrowheads). Yellow arrows indicate MTJs. (E) XZ image of perinuclear and membrane staining of Dlar at membrane-associated Z-discs (white arrowhead). Anterior is left in all images.

Larval body wall muscles are established during embryogenesis and persist throughout larval development [45]. Phalloidin labels filamentous actin (F-actin) in contractile muscles (**Fig 2A and 2C**) and is enriched at the Z-disc (**Fig 2D,** white arrowheads), a structure that defines the borders of each sarcomere and serves as an anchor for thin filament proteins [46]. Since Dlar is present in the developing musculature (**Fig 1E**), we next examined the subcellular distribution of Dlar protein in contractile third larval instar (L3) body wall muscles. First, we generated an antibody against the Dlar fibronectin domain 5 (FN5) that recognized overexpressed Dlar protein (**Fig 2B**, left panel). Immunoreactivity was decreased in *Dlar RNAi* knockdown larvae compared to control larvae (**Fig 2B**, right panel and quantification). A staining pattern that partially overlapped with F-actin was found via immunofluorescence using anti-Dlar FN5 (**Fig 2C1 and 2D2**). Importantly, the majority of Dlar protein could be found specifically at the sarcolemma at a site consistent with the costamere (**Fig 2E**, white arrowheads), a transmembrane complex of proteins that physically connects the sarcolemma to the internal muscle cytoskeleton at repetitive Z-disc structures [31].

### RNAi knockdown of Dlar affects actin patterning and integrin localization

Since the reduction of Dlar in muscle tissue is pupal lethal (**Fig 1F**), we examined L3 larvae just prior to pupation to assess if a reduction of this RPTP affects muscle morphology. Both the transcript (**S1 Fig**) and protein (**Fig 2B**) levels of Dlar were reduced upon induction of Dlar RNAi, thus justifying this knockdown approach for phenotypic analysis. The stereotypical pattern of 30 abdominal muscles per hemisegment appeared normal, including intact muscle attachments, in *WT* or *24B>Dlar RNAi* muscles (**S1 Fig**). Closer examination revealed sarcomeric patterning defects (dotted lines) and a decrease in the overall muscle integrity, occasionally with enlarged spaces between adjacent myofibrils (white arrowheads).

**Fig 3:**
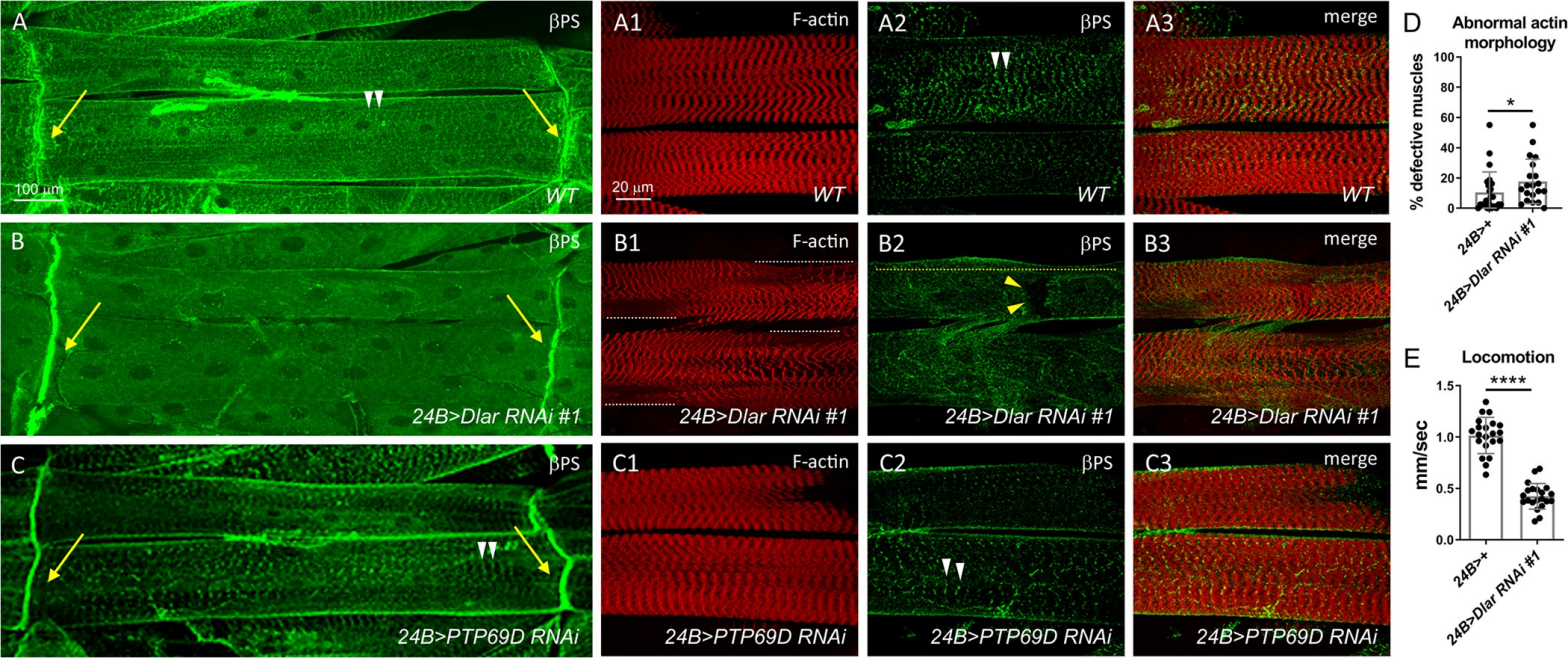
Dlar is required for the localization βPS integrin at costameres. Low (A-C) or high (A1-A3, B1-B3, C1-C3) magnification images of ventral longitudinal muscles in the L3 stage. Anterior is left in all images. WT (A), 24B>Dlar RNAi #1 (B), and 24B>PTP69D RNAi (C) larvae display characteristic βPS localization by immunofluorescence at the MTJ (yellow arrows). F-actin patterning and Z-disc co-localization of βPS appears normal in WT (A-A3) and PTP69D RNAi muscles (C-C3). (B-B3) Induction of Dlar RNAi results in a loss of sarcomeric patterning (white dotted line) and a general loss of βPS distribution at Z-disc sites across muscle (yellow dotted line). Disruption of the sarcolemmal integrity was also more pronounced in Dlar RNAi muscles (B2, yellow arrowheads). (D) Bar and scatter plot depicts that the percentage of defective muscles assayed by F-actin staining is increased upon induction of Dlar RNAi in muscle tissue. Mean +/- SD. *, p< 0.05. N ≥ 20. (E) Locomotion assay shows that a reduction in Dlar compromises movement of L3 larvae as depicted in the bar and scatter plot. Mean +/- SD. ****, p< 0.001. N ≥ 20.

Integrin adhesion complexes at the *Drosophila* larval costamere are comprised of αPS2/βPS heterodimers and associated cytosolic proteins that link the ECM to the internal actin cytoskeleton [31]. To assess the effect of Dlar depletion on integrin localization at costameres, we utilized immunofluorescence to visualize βPS protein in *Dlar RNAi* muscles. Integrin βPS is normally found at the myotendinous junction (MTJ) (**Fig 3A**, yellow arrows) and at costamere sites that align with Z-discs (**Fig 3A and 3A3**, white arrowheads) in *WT* larval muscle [31]. Dlar knockdown larvae displayed normal accumulation of βPS at the MTJ (**Fig 3B**, yellow arrows), which is consistent with the absence of Dlar protein at this location (**Fig 2C1**, yellow arrows). However, the costameric association of βPS was lost and instead broadly distributed across the sarcolemma in *24B>Dlar RNAi* muscles (**Fig 3B and 3B3**, yellow dotted line). Analysis and quantification of actin patterning abnormalities (white dotted lines in **Fig 3B1**) revealed a small, but significant difference in *Dlar RNAi* muscles compared to *24B-Gal4* controls (**Fig 3D**). To determine the functional significance of the observed integrin mislocalization and sarcomeric disorganization phenotypes, we utilized a locomotion assay to measure the speed of crawling larvae. In this context, we found that Dlar knockdown in muscle tissue greatly reduced the ability of larvae to traverse across agar plates compared to *WT* controls (**Fig 3E**). These actin and integrin defects were caused by a specific decrease in Dlar as *mef2>Ptp69D RNAi* muscles exhibited normal integrin distribution and F-actin patterning (**Fig 3C and 3C3**, yellow arrows and white arrowheads).

**Fig 4:**
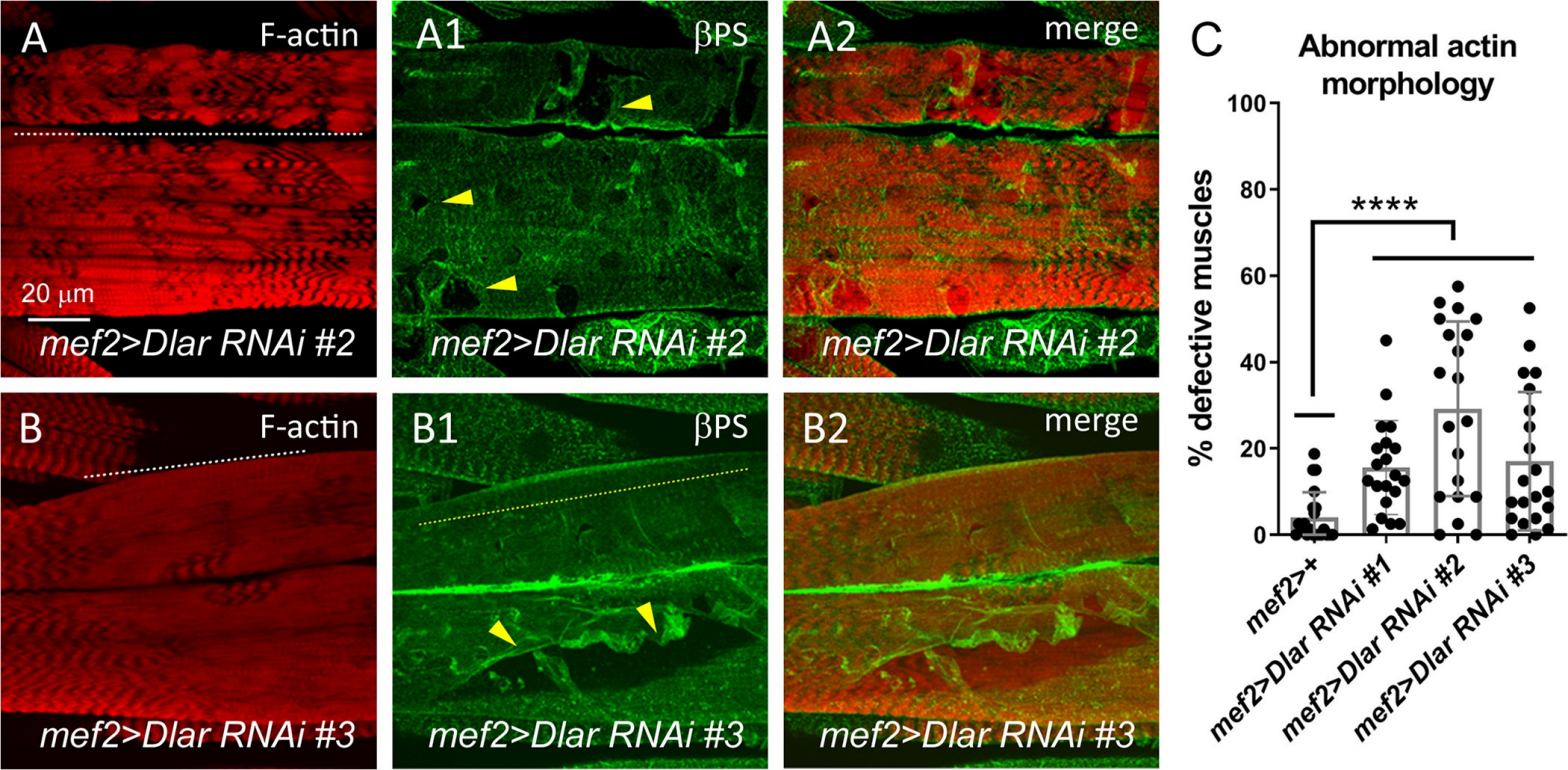
βPS is mislocalized in additional Dlar RNAi lines. (A-B2) Confocal micrographs of L3 ventral longitudinal muscles. Anterior is left in all images. F-actin (A,B) or βPS integrin (A1,B1) staining in mef2>Dlar RNAi #2 (A-A2) or mef2>Dlar RNAi #3 (B-B2) muscles reveal a general loss of sarcomeric patterning (white dotted line) and integrin mislocalization (yellow dotted line). The integrity of the sarcolemma is also disrupted in both RNAi lines (A1,B1, yellow arrowheads). (C) Bar and scatter chart showing the percentage of defective muscles. Mean +/- SD. ****, p< 0.01. N ≥ 20.

To further substantiate a role for Dlar in F-actin patterning and βPS integrin localization and to control for potential off-target effects of *Dlar RNAi*, we examined the larval muscles of two additional UAS-*Dlar RNAi* lines using the alternative muscle *mef2*-GAL4 driver. Phalloidin staining of muscles in Dlar-reduced larvae showed hypercontraction indicated by a shortening of sarcomeres (**Fig 4A and 4B**, white dotted lines), a lack of Z-disc associated βPS integrin striations (**Fig 4B1**, yellow dotted line), and numerous tears in the sarcolemmal membrane (**Figs 3B2, 4A1 and 4B1**, yellow arrowheads). Qualitatively, these *mef2*-induced defects appeared stronger than those observed with *24B*-Gal4. Indeed, quantitation of F-actin stained muscles revealed an increase in *Dlar RNAi* muscles using the *mef2* driver (**Fig 4C**). Together, these data shows that a reduction in Dlar correlates with sarcomeric patterning defects, a redistribution of βPS protein across the sarcolemma, and a loss of functional muscle contraction.

### Structural analyses of FN fragments from type IIa RPTPs

Strikingly, our data substantiate a functional role for Dlar in focal adhesion (FA) complexes in muscle tissue. Skeletal muscle fibers form two distinct FA complexes, one at the MTJ and the other at the costamere, and are comprised of integrin dimers that link the ECM to the actin cytoskeleton [47]. The presence of Dlar at costameres is consistent with data whereby mammalian LAR localizes to adhesion complexes to regulate the actin cytoskeleton [48–50]. Additionally, on the basal surface of the *Drosophila* follicular epithelium, *Dlar* or *mys* (encodes for βPS) mutant clones display a loss of F-actin polarity and βPS integrin mislocalization that causes a ‘round egg’ phenotype [51]. Decreasing the *mys* gene dosage by half in a *Dlar* null background increases the penetrance of this phenotype, suggesting that these two genes functionally interact. Moreover, the Dlar fragments sufficient to rescue this aberrant actin polarity phenotype in a *Dlar*-null background were narrowed to domains FN4-FN6 in the extracellular region of Dlar [52]. Given these findings and combined with our observation that a decrease of Dlar in larval muscle causes integrin mislocalization and actin patterning abnormalities, we undertook a bioinformatics approach to scan for sequence elements that may mediate an interaction with the extracellular region of integrins.

**Fig 5:**
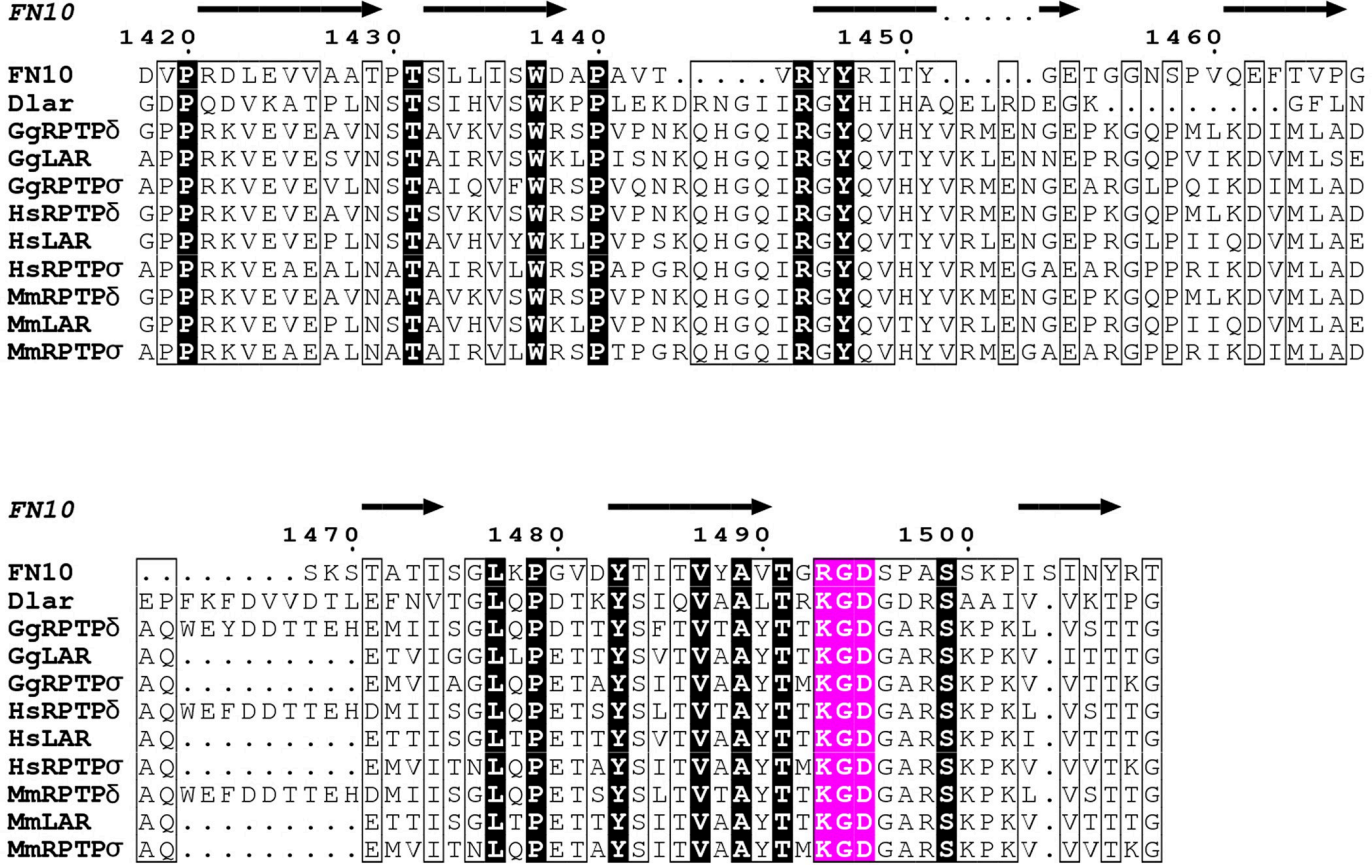
Sequence alignment depicting conservation between the RGD peptide found in domain FN10 of human fibronectin and in the FN5 domains of type IIa RPTPs. The secondary structure element above the structure corresponds to those found in FN10 [57]. Strictly conserved residues are shown in black while the RGD and KGD residues highlighted in magenta. Sequences for the FN5 domains of type IIa RPTPs are from chicken (Gg), human (Hs), and mouse (Mm). The figure was prepared using ESPRIPT [58].

The adhesive function of a tripeptide sequence corresponding to Arg-Gly-Asp (RGD) was first discovered in the ECM protein, human fibronectin (FN) [53, 54]. This RGD sequence defines an integrin-binding motif and is found in the 10^th^ FN repeat of human fibronectin (FN10). Surprisingly, we uncovered a tripeptide, Lys-Gly-Asp (KGD), in the Dlar FN5 domain that is similar to the RGD motif in FN10 and is conserved in the Lar-RPTP homologs across species (**Fig 5**). This motif is also present in the *Drosophila* integrin ECM ligand thrombospondin found at muscle attachment sites [55], and KGD motifs have been used in the design of small integrin antagonists [56]. Thus, we hypothesized that the FN5 domain of Dlar may directly interact with integrin complexes via this conserved KGD tripeptide motif.

**Fig 6:**
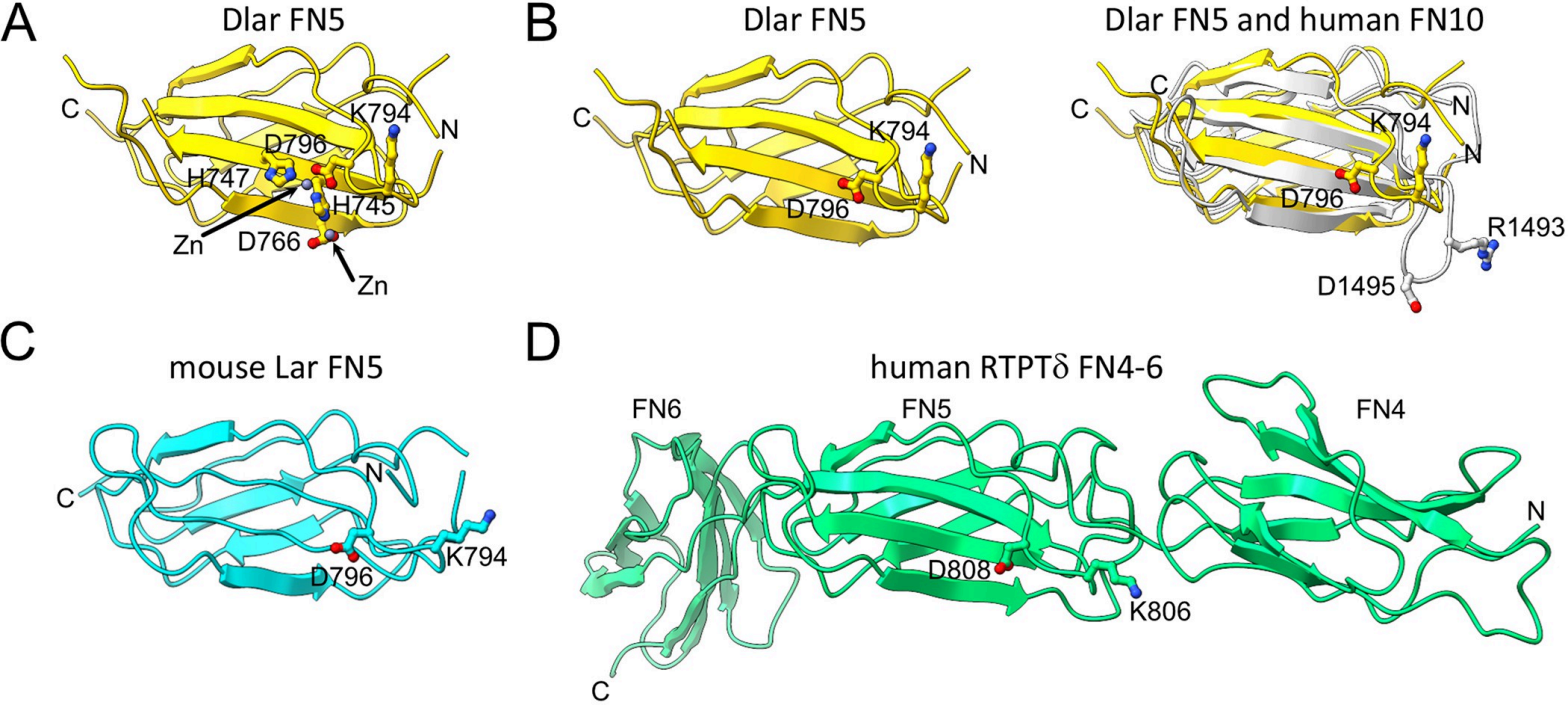
Crystal structures of Dlar and vertebrate orthologues showing the positions of the KGD tripeptide. (A) Ribbon diagram depicting the structure of the FN5 domain of Dlar (yellow). The lysine (K794) and aspartate (D796) residues of the KGD tripeptide are shown in ball-and-stick representation with nitrogen and oxygen atoms colored blue and red, respectively. The two zinc ions bound to Dlar FN5 are shown as gray spheres, and Zn^2+^-binding residues H745, H747, and D766 are shown in ball-and-stick representation. (B) A ribbon diagram of DlarFN5 is shown on the left without the Zn^2+^ ions and Zn^2+^-binding residues for the sake of clarity. On the right, this domain is superimposed onto the tenth FN repeat of human fibronectin (white, PDB ID 1FNF). The arginine and aspartate residues of the RGD tripeptide are shown in ball-and-stick representation. Note that these residues are found in a loop that extends away from the FN domain unlike the position of the KGD motif in Dlar. (C) A ribbon diagram of the FN5 domain of mouse Lar is shown in cyan. Residues in the mouse Lar KGD motif are shown in ball-and-stick representation. (D) The crystal structure of domains FN4-FN6 of human RPTPδ is shown in a ribbon representation and colored green. The lysine and aspartate residues found in the KGD motif are shown in ball-and-stick representation. In all panels, the letters N and C indicate the positions of the N and C termini, respectively.

To determine if Dlar possesses the structural characteristics of an integrin ligand, we sought to find a crystallizable region of the Dlar extracellular region encompassing the FN5 domain. The following constructs were overexpressed in *E*. *coli*: FN4-FN7, FN4-FN6, FN4-FN5, FN5-FN6 and FN5. Proteins including the FN4-FN6, FN4-FN5, and FN5 were soluble, but only the FN5 yielded diffraction quality crystals (**Tables 1 and 2**). Interestingly, a ~5 molar equivalent of Zn^2+^ was required to grow diffraction-quality crystals of Dlar FN5. We used the anomalous signal from bound Zn^2+^ ions to calculate experimental phases and obtain an initial model. The final structure was refined to a resolution of 1.3 Å. Overall, the Dlar FN5 adopts a prototypical β-sandwich fold described for FN domains with two Zn^2+^ ions (**Fig 6A**). The first Zn^2+^ is bound by His-745 in strand C and Asp-766 at a crystal contact and may be an artifact of crystallization. The second is coordinated by residues within a single FN5 domain, including the Asp-796 from the KGD motif and two histidine residues, His-745 and His-747.

**Table 2:** Crystallographic data and refinement statistics.

Data collection			
Crystal	Drosophila Lar FN5	Mouse LAR FN5	Human RPTPδ FN4-FN6
PDB accession code	6X38	6X39	6X3A
Wavelength (Å)	1.00	1.00	1.00
Resolution range (Å)	50.00–1.30 (1.35–1.30)	50.00–1.70 (1.76–1.70)	54.60–1.77 (1.80–1.77)
Space group	P 2_1_ 2_1_ 2_1_	P 3_2_ 2 1	I 2
Cell dimensions			
a, b, c (Å)	40.580, 42.658, 51.347	86.110, 86.110, 29.344	95.060, 34.570, 109.199
α, β, γ (°)	90.00, 90.00, 90.00	90.00, 90.00, 120.00	90.00, 90.33, 90.00
Unique reflections	22,169	13,907	35,136
Multiplicity	13.0 (9.4)	21.3 (15.1)	7.2 (6.2)
Completeness (%)	98.4 (96.1)	99.5 (97.6)	99.5 (98.7)
I/σI	12.7 (6.6)	27.7 (4.2)	13.5 (2.4)
Rmerge	0.115 (0.290)	0.119 (0.492)	0.077 (0.679)
**SAD Phasing at 1.3 Å**			
Zn sites	2		
BAYES-CC	39.9 ± 22.5		
FOM from SAD	0.53		
**Refinement**			
Resolution range (Å)	32.81–1.30	28.19–1.70	34.06–1.77
Reflections used in refinement	20,096	12,371	33,136
Reflections used for R_free_	2,004	1,390	1,997
R_work_/R_free_	0.167/0.182	0.164/0.190	0.195/0.239
No. of non-hydrogen atoms	845	888	2,745
Protein	733	753	2,427
Solvent	110	123	312
Ligand/ion	2	12	6
Root mean square deviations			
Ideal bonds (Å)	0.010	0.011	0.007
Ideal angles (°)	0.920	1.154	0.824
Average B factors (Å^2^)	15.9	22.6	38.0
Protein	14.6	21.7	37.8
Water	24.8	28.3	39.1
Ligand/ion	9.0	21.9	70.2
Ramachandran statistics			
Favored (%)	97.7	96.9	97.0
Allowed (%)	2.3	3.1	3.0
Rotamer outlier (%)	1.2	0.0	0.4

This arrangement differs from the RGD tripeptide located within the tenth FN repeat of human fibronectin [57]. The Dlar FN5 domain and the FN10 share 25% sequence identity at the amino acid level and sequence alignment reveals that the K/RGD motif is located in a similar position in both proteins (**Fig 5**). The Arg-1493 and Asp-1495 side chains of FN10 are located in the solvent-exposed region of the flexible loop (**Fig 6B**, right panel). Although the KGD of Dlar lies in the same general area, the side chains are not as accessible for binding with the Asp-796 positioned against the β-strand and the Lys-794 pointing back toward the molecule instead of accessible in the solvent (**Fig 6A**). Importantly, the KGD does not extend away from the domain as seen in FN10 (**Fig 6B**).

We sought to gain further insights into the position of the conserved KGD motif in mammalian homologues of Dlar. We thus determined the crystal structures of the FN5 domain of mouse LAR and the FN4-FN6 fragment of human RPTPδ (**Tables 1 and 2**). The LAR FN5 crystal structure was solved by molecular replacement using Dlar FN5 as the model and refined to a resolution of 1.7 Å (**Fig 6C**), while the structure of the FN4-FN6 fragment was to a resolution of 1.77 Å (**Fig 6D**). Overall, the LAR FN5 adopts a structure that is very similar to that of Dlar FN5. However, the KGD peptide remains poorly accessible and is thus unlikely to favor binding to integrin complexes. A similar conclusion can be drawn from examining the position of the KGD sequence within the structure of RPTPδ FN4-FN6 (**Fig 6D**). The three-domain fragment adopts an extended conformation, but the KGD sequence lies at the interface between domains FN4 and FN5, which makes the tripeptide unavailable for binding to integrins. Thus, if Dlar and its mammalian homologues interact directly with integrins, they will use a mechanism that is distinct from the canonical RGD/integrin binding interaction.

### Identification of potential binding partners for LAR-RPTPs

Structural analyses of the predicted potential integrin-binding sequence in the FN5 domains of Dlar, mouse LAR, and human RPTPδ indicated that the KGD tripeptide does not adopt the structural characteristics of a bona-fide integrin-binding motif (**Fig 6**). Despite these findings, there are distinct regions capable of participating in other protein-protein interactions. The mammalian RPTPs associate with multiple physiologically relevant binding partners through interactions within their Ig1-Ig3 [59], FN1-FN2 [10], and FN5 [9] domains and some interactions require both the Ig and FNIII regions [60]. Additionally, the Dlar FN4-6 and the Dlar FN7-9 domains have been implicated in maintenance of epithelial polarity and R-cell targeting in the *Drosophila* nervous system [51, 61]. Hence, we undertook a comprehensive effort to determine whether the identification of extracellular interactions may provide an explanation for the integrin mislocalization phenotype in Dlar-knockdown muscles.

To detect potential binding partners of Lar-RPTPs, we performed pull-down experiments with distinct regions of *Drosophila* or mammalian homologues of Dlar. Purified fragments corresponding to Dlar Ig1-Ig2, Dlar FN4-FN5, or the entire ectodomain of soluble Dlar (sDlar) were separately attached to NHS-Activated Sepharose, incubated with L3 larval lysates, and the resulting bound protein complexes were subjected to MS analysis for peptide identification. NHS-Sepharose or protein A agarose served as negative controls (**S1 Table**). With a statistical cutoff of p < 0.05, the individual number of proteins that emerged from each experiment ranged from 30–252 (**S2 Fig**). Notably, each individual pulldown resulted in the successful identification of enriched Dlar peptides (**S3 Table, S1 Appendix**), taken as a positive control of Dlar ectodomain dimerization [62, 63].

The identification of three heparan sulfate proteoglycans (HSPGs) in the Dlar Ig1-Ig2 pull-downs validate our overall approach, as binding sites for heparan sulfate chains in the N-terminal Ig domain of Dlar have been reported previously [59, 64]. First, Syndecan (Sdc) is a known Dlar ligand that associates with Dlar during neuromuscular junction (NMJ) formation [65]. The next HSPG is a member of the glypican family called Division abnormally delayed (Dally) [66]. There are only two glypicans in flies and the other, Dallylike (dlp), is another *in vivo* Dlar ligand and has been implicated in NMJ formation with Sdc [64]. Last, a secreted HSPG that is incorporated into BMs, terribly reduced optic lobes (Trol), is the homolog to the mammalian proteoglycan Perlecan (Pcan) [67]. A possible link exists between our identification of Wnt/Wingless (Wg) and Trol, since these proteins are implicated in the bidirectional regulation of NMJ maturation [68]. An additional connection to Wnt/Wg signaling was uncovered through the identification of a non-HSPG protein identified in the Dlar Ig1-Ig2 pull-down called Secreted Wg-interacting molecule (Swim), a secreted lipocalin [69].

Consistent with the hypothesis that Dlar binds to BM proteins in muscle, additional Pcan binding partners include Laminin and Nidogen [70], Fibronectin [71], integrins [72] and Dystroglycan (Dg) [73]. In an effort to broaden our understanding of BM protein complexes, we performed pull-down assays using Fc fusions of the BM protein Glutactin (Glt) (**S3 Table, S1 Appendix**). Notably, we identified peptides corresponding to two subunits of Collagen IV (Gc25C and Vkg) as well as Dlar. Note that we did not uncover Glt as an interacting protein in the reciprocal Dlar pulldowns. Finally, this proteomic analysis also highlighted potential interactions with Cd98Hc, a subunit of a conserved amino acid transporter which also functions in cell fusion and cell adhesion [74]. Cd98 is a transmembrane glycoprotein that can regulate integrin activation by binding to βPS tails through the Cd98Hc subunit possibly providing the physical link between integrins and Dlar through a tripartite complex or integrin signaling pathway [75, 76].

To identify conserved ligands for mammalian LAR FN4-FN6 domains, affinity isolation experiments were carried out from the following mammalian cell line lysates: B35 (rat neuroblastoma), C2C12 (mouse myoblast), C6 (rat glioblastoma), HEK293 (human embryonic kidney), and Neuro2a (mouse neuroblastoma). Lysates were incubated with the following purified bait proteins attached to NHS-resin: the FN4-FN7 domains of mouse Lar (Mlar FN4-FN7) and mouse RPTPδ (MRPTPD FN4-FN7). The FN1-FN3 domains of mouse CNTN1 (mCNTN1 FN1-FN3) or mouse CNTN6 (mCNTN6 FN1-FN3) served as negative controls (**S2 Table**). Depending on the cell line that was utilized for pulldowns, the number of LAR or PTRPD proteins ranged from 38–514 or 96–731, respectively (**S3 Fig**).

MS analysis resulted in the identification of two BM proteins, collagen α1 chain (Col1a1) from the Lar/C2C12 experiment and Perlecan (hspg2) from the Lar/C2C12, RPTPδ/C2C12 and Lar/Neuro2a pull-downs (**S4 Table, S2 Appendix**). An additional protein uncovered was the non-integrin transmembrane receptor of the costamere, Dystroglycan (Dg), from both Lar and RPTPδ/C6 cells. Dg is implicated in muscular dystrophies and has roles in neural development as well [77]. Integrin subunits were present in both LAR and PTRPδ pull-downs. However, these proteins showed low enrichment and were not identified in C2C12 muscle cells. Validation has not been undertaken for MS identifications from the mammalian cell line pull-downs but supports the notion that the Dlar family of proteins may physically interact with BM proteins in muscle tissue.

### Interplay between PRTP-ECM-integrin complexes

Extensive studies on LAR-RPTP interactions and their extracellular ligands in neuronal tissue may provide context for our observations that Dlar can physically associate with BM proteins. Individual mammalian LAR-RPTP family members (LAR, RPTPδ, or RPTPσ) are localized to distinct excitatory and/or inhibitory synapses, presumably to promote presynaptic assembly and to maintain physical interactions with extracellular binding proteins at postsynaptic complexes [78, 79]. Likewise, local Dlar-BM interactions may promote the organization of muscle tissue with the surrounding milieu, whereby Dlar interacts with BM proteins to provide support for the underlying tissue. When BMs are defective, the adjacent layers lose integrity. Consistent with this hypothesis, muscle-specific knockdown of the BM protein Glutactin (Glt) resulted in a loss of βPS and Dlar protein localization at the costamere (**S4 Fig**). In this model, defective association between Dlar and its BM partners leads to cytoskeletal reorganization. These rearrangements would not favor integrins adopting a high-affinity binding conformation, which would result in loss of integrity in muscle tissues.

An additional, or alternative, role for LAR-RPTP complexes as a signaling hub would allow for the coordination of multiple proteins within a complex and provide a mechanism to titrate activity [80, 81]. For example, netrin-G ligand-3 (NGL-3) and neurotrophin receptor tropomyosin-related kinase C (TrkC) can simultaneously bind to RPTPσ, while NGL-3 also interacts with LAR and RPTPσ at synapses. These multiple interactions may allow for either cooperation or competition between binding pairs and influence intracellular signaling events. Neuronal ECM environments rich in chondroitin sulfate (CS) surround either developing or regenerating axons and evidence suggests that CS-RPTPσ or chondroitin sulfate proteoglycan (CSPG)-LAR complexes prevent neuronal regeneration after injury [82–84]. HSPGs bind to and influence RPTP activity, including Dlar function in NMJ development or in mammalian neuron synapse formation and plasticity [64, 85]. Since integrins are expressed at synapses and integrin ligands are found in the extracellular environment, CSPG/HSPG interactions with LAR-RPTP complexes may indirectly affect integrin signaling [86]. Thus, if the observed MS interactions with HSPGs in larval lysates influence the binding and/or activity of Dlar during normal muscle use, BM interactions with integrins may be affected.

### Conclusions and limitations of this study

The most striking finding of the work presented here is that the Dlar receptor phosphatase localizes to costameres of *Drosophila* larval muscles. This result expands upon published literature that suggests Dlar is almost exclusively found in neural tissues. Furthermore, knocking down the expression of Dlar using a muscle-specific driver results in decreased larval locomotor activity as well as mislocalization of costameric integrin subunits. Thus, these experiments suggest that Dlar plays a role in the maintenance of the *Drosophila* musculature and may intersect with integrin localization and/or signaling.

Initially we hypothesized that Dlar and integrins may physically interact based on the presence of a conserved integrin-binding like motif in the FN5 repeat of the type IIa RPTPs. However, our structural analyses did not show an accessible KGD sequence for integrin binding as in the canonical RGD peptide in FN10 of fibronectin. Further extensive proteomic analyses did not hint at any physical interaction between integrins and Dlar or any mammalian counterparts. Thus, we think it extremely unlikely that Dlar and integrins interact directly with one another, leaving open the question of the striking integrin mislocalization we observed in *dlar* mutants. However, we identified several BM proteins throughout these analyses suggesting that Dlar, as well as LAR and RPTPδ, might associate with some of these matrix proteins. We thus hypothesize that the disruption of the BM in animals in which expression of Dlar is repressed can account for the muscle phenotypes.

## Supporting information

S1 Table *Drosophila* LC-MS/MS experimental index. (DOCX)

S2 Table Mammalian cell culture LC-MS/MS experimental index. (DOCX)

S3 Table Proteins identified in LC-MS/MS that co-purify with *Drosophila* Dlar or Glt baits. (DOCX)

S4 Table Proteins identified in LC-MS/MS that co-purify with Mammalian PTPRD or LAR baits. (DOCX)

S1 Fig Dlar is required for larval sarcomeric patterning and muscle integrity. (A) Bar graph depicting relative *dlar* transcript levels in control (da>+) or knockdown (da>Dlar RNAi #1) larvae. Mean +/- SD. ****, p< 0.001. N = 3 biological replicates and 3 technical replicates for each genotype. (B-D) Immunofluorescence of F-actin in L3 muscle. Left is anterior in all images. (B) Two complete hemisegments of WT musculature. White dashed line outlines a single hemisegment. (C) Overall muscle patterning is normal *in 24B>Dlar RNAi #1* larvae. White dashed line outlines a single hemisegment. (D) Composite image of six hemisegments of 24B>Dlar RNAi #1 muscles. Sarcomeric patterning defects (white dashed line), splits in myofibrils (arrowhead), broken or tearing muscle (boxes), increase in distance between adjacent dorsal myofibers (solid braces) can be observed.
(TIF)

S2 Fig Volcano plots for Dlar pulldown experiments. (A-G) Volcano scatterplots showing the relative number of proteins identified in individual Dlar pulldown experiments. Y-axis shows statistical significance (p<o.o5) and x-axis depicts fold change.
(TIF)

S3 Fig Volcano plots for mammalian LAR and PTRPS pulldown experiments. (A,B) Volcano scatterplots showing the relative number of proteins identified in individual pulldown experiments in the indicated cell types. Y-axis shows statistical significance (p<o.o5) and x-axis depicts fold change.
(TIF)

S4 Fig βPS and Dlar are mislocalized in *Glt* knockdown muscle. (A-D2) Immunofluorescence of βPS integrin (A-B2, green) or Dlar (C-D2, green) in *WT* (A-A2,C-C2) or *24B>Glt RNAi* (B-B2,D-D2) co-stained with phalloidin (red) in L3 larval muscle. White arrowheads indicate βPS striations. Yellow arrowheads show regions of torn or damaged sarcolemma. Yellow dotted line highlight regions of abnormal patterning. (E) Bar graph depicting relative *Glt* transcript levels in control (*da>+*) or knockdown (*da>Glt RNAi*) larvae. Mean +/- SD. **, p< 0.01. N = 3 biological replicates and 3 technical replicates for each genotype. (F,G) Scatter and bar graphs representing abnormal muscle defects assayed by F-actin patterning (F) or locomotor ability of L3 larvae (G) in *WT* or *24B>Glt RNAi*. Mean +/- SD. ****, p<0.001; **, p< 0.01. N ≥ 20.
(TIF)

S1 Raw images Raw Western blot images. Original, uncropped scans for Western blots in Fig 2B. Boxed rectangle in each blot corresponds to the lanes of cropped regions in Fig 2B, while the ‘X’ indicates biological replicates. Whole larvae were homogenized in SDS sample buffer, boiled 10 minutes, centrifuged to remove debris, and run on a 7% Tris-Glycine SDS-PAGE gel. (A) UAS-based overexpression of Dlar in muscle tissue using *mef2*-Gal4. (B) Knockdown of Dlar RNAi in all tissues using *da*-Gal4. ‘2x’ under blot indicates that twice the amount of *da>lacZ* lysate was loaded into this lane compared to other *da>lacZ* control lanes.
(TIF)

S1 Appendix Raw Perseus data for LC-MS/MS with *Drosophila* Dlar or Glt baits. (XLSX)

S2 Appendix Raw Perseus data LC-MS/MS with Mammalian PTPRD or LAR baits. (XLSX)
