# Supplementary material for: Complex protein interactions mediate Drosophila Lar function in muscle tissue
Source: PLoS One. 2022 May 27;17(5):e0269037. doi: 10.1371/journal.pone.0269037 (PMC9140312; doi:10.1371/journal.pone.0269037)
Supplement: S1 Table — (DOCX) [file pone.0269037.s001.docx]

**S1 Table. *Drosophila* LC-MS/MS experimental index.**

| **Experiment** | **Pre-clear** | **Control** | **Bait** | **Bait** | **Buffer** |
| --- | --- | --- | --- | --- | --- |
| 1491a | NHS- sepharose | NHS-Rb IgG | NHS-anti-  Dlar Ig12 |  | 50mM Tris 7.5, 150mM NaCl, 10% (v/v) glycerol, 0.1% (w/v) Na deoxycholate, 0.1% (v/v) TritonX-100, 1mM CaCl2, 1mM MgCl2, 100uM Zn(OAc)_2_ |
| 1454a | NHS-sepharose | NHS-Rb IgG | NHS-anti-  Dlar Ig12 |  | 50mM Tris 7.5, 150mM NaCl, 1% (v/v) TritonX-100, 1mM CaCl_2_, 1mM MgCl_2_, 100uM Zn(OAc)_2_ |
| 1454b | NHS- sepharose | NHS-Rb IgG | NHS-anti-  Dlar Ig12 |  | 50mM Tris 7.5, 150mM NaCl, 1% (v/v) TritonX-100, 1mM CaCl_2_, 1mM MgCl_2_, 100uM Zn(OAc)_2_ |
| 1454c | NHS- sepharose | NHS-Rb IgG (from 1454a) | NHS-  Dlar Ig12 | NHS-  Dlar FN45 | 50mM Tris 7.5, 150mM NaCl, 1% (v/v) TritonX-100, 1mM CaCl_2_, 1mM MgCl_2_, 100uM Zn(OAc)_2_ |
| 1491b | NHS- sepharose | NHS-CNTN4 FN1-3 | NHS-  Dlar FN45 |  | 50mM Tris 7.5, 150mM NaCl, 1% (v/v) TritonX-100, 1mM CaCl_2_, 1mM MgCl_2_, 100uM Zn(OAc)_2_ |
| 1491c | NHS- sepharose | NHS-Rb IgG | NHS-sDlar |  | 50mM Tris 7.5, 150mM NaCl, 1% (v/v) TritonX-100, 1mM CaCl_2_, 1mM MgCl_2_, 100uM Zn(OAc)_2_ |
| 1456 | protein A agarose | Fc | sDlar-Fc | Glt-Fc | 50mM Tris 7.5, 150mM NaCl, 1% (v/v) TritonX-100, 1mM EDTA, 1mM EGTA |
