# Supplementary material for: Complex protein interactions mediate Drosophila Lar function in muscle tissue
Source: PLoS One. 2022 May 27;17(5):e0269037. doi: 10.1371/journal.pone.0269037 (PMC9140312; doi:10.1371/journal.pone.0269037)
Supplement: S2 Table — (DOCX) [file pone.0269037.s002.docx]

**S2 Table. Mammalian cell culture** **LC-MS/MS experimental index.**

| **Cell culture** | **Pre-clear** | **Control** | **Control** | **Bait** | **Bait** | **Buffer** |
| --- | --- | --- | --- | --- | --- | --- |
| C6 | CNBr- sepharose | CNTN1 Ig1-3 | CNTN6 Ig1-3 | Mlar FN4-7 | MPTPRD FN4-7 | 50mM Tris 7.5, 150mM NaCl, 1% (v/v) TritonX-100, 1mM CaCl_2_, 1mM MgCl_2_ |
| B35 | CNBr- sepharose | CNTN1 Ig1-3 | CNTN6 Ig1-3 | Mlar FN4-7 | MPTPRD FN4-7 | 50mM Tris 7.5, 150mM NaCl, 1% (v/v) TritonX-100, 1mM CaCl_2_, 1mM MgCl_2_ |
| C2C12 | CNBr- sepharose | CNTN1 Ig1-3 | CNTN6 Ig1-3 | Mlar FN4-7 | MPTPRD FN4-7 | 50mM Tris 7.5, 150mM NaCl, 1% (v/v) TritonX-100, 1mM CaCl_2_, 1mM MgCl_2_ |
| N2A | CNBr- sepharose | CNTN1 Ig1-3 | CNTN6 Ig1-3 | Mlar FN4-7 | MPTPRD FN4-7 | 50mM Tris 7.5, 150mM NaCl, 1% (v/v) TritonX-100, 1mM CaCl_2_, 1mM MgCl_2_ |
| HEK293 | CNBr- sepharose | CNTN1 Ig1-3 | CNTN6 Ig1-3 | Mlar FN4-7 | MPTPRD FN4-7 | 50mM Tris 7.5, 150mM NaCl, 1% (v/v) TritonX-100, 1mM CaCl_2_, 1mM MgCl_2_ |
