# Supplementary material for: Complex protein interactions mediate Drosophila Lar function in muscle tissue
Source: PLoS One. 2022 May 27;17(5):e0269037. doi: 10.1371/journal.pone.0269037 (PMC9140312; doi:10.1371/journal.pone.0269037)
Supplement: S3 Table — (DOCX) [file pone.0269037.s003.docx]

**S3 Table. Proteins identified in LC-MS/MS that co-purify with *Drosophila* Dlar or Glt baits.**

| **Enrichment^1^** | **Sample intensity (log2)** | **Control Intensity (Log2)** | **Bait vs. Control** | ***Drosophila* protein^2,3^** |
| --- | --- | --- | --- | --- |
| 3,378 | 28.15678 | 20.03162 | sDlar vs RbIgG | Alp1 |
| 2,091 | 28.97246 | 21.32706 | sDlar vs RbIgG LFQ | Alp1 |
| 860 | 26.78905 | 20.03162 | anti-Dlar vs RbIgG | Alp1 |
| 1,500 | 28.64031 | 21.32706 | anti-Dlar vs RbIgG LFQ | Alp1 |
| 615 | 26.22823 | 19.80595 | DlarIg12 vs RbIgG | Alp1 |
| 11,511 | 26.18573 | 16.83467 | DlarIg12 vs RbIgG LFQ | Alp1 |
| 566 | 26.14418 | 19.80595 | DlarFN45 vs RbIgG | Alp1 |
| 7,328 | 25.73412 | 16.83467 | DlarFN45 vs RbIgG LFQ | Alp1 |
| 21 | 21.46219 | 18.43224 | anti-Dlar vs RbIgG | alpha-Est2 |
| 12 | 21.04434 | 18.52814 | anti-Dlar vs RbIgG | alpha-Est4 |
| 24 | 21.74526 | 18.55659 | sDlar vs RbIgG | Caix |
| 19 | 19.23932 | 16.27402 | Glt-Fc vs Fc | Caix |
| **23** | **22.18997** | **19.05711** | **sDlar vs RbIgG** | **CD98hc** |
| **47** | **22.91561** | **19.05711** | **anti-Dlar vs RbIgG** | **CD98hc** |
| 339 | 24.50996 | 18.68266 | sDlar vs RbIgG | CG10211 |
| 60 | 25.63362 | 21.54656 | sDlar vs RbIgG LFQ | CG10211 |
| 302 | 24.39316 | 18.68266 | anti-Dlar vs RbIgG | CG10211 |
| 70 | 25.80154 | 21.54656 | anti-Dlar vs RbIgG LFQ | CG10211 |
| 86 | 21.13333 | 16.67632 | sDlar-Fc vs Fc | CG13492 |
| 17 | 20.9239 | 18.08106 | sDlar vs RbIgG | CG1371 |
| 101 | 26.37013 | 21.75397 | sDlar vs RbIgG | CG2233 |
| 19 | 27.27293 | 24.32065 | sDlar vs RbIgG LFQ | CG2233 |
| 38 | 25.3981 | 21.75397 | anti-Dlar vs RbIgG | CG2233 |
| 69 | 25.00599 | 20.77828 | DlarIg12 vs RbIgG | CG2233 |
| 85 | 25.07545 | 20.6344 | DlarIg12 vs RbIgG LFQ | CG2233 |
| 36 | 24.34881 | 20.77828 | DlarFN45 vs RbIgG | CG2233 |
| 13 | 23.23427 | 20.6344 | DlarFN45 vs RbIgG LFQ | CG2233 |
| 15 | 27.02179 | 24.32065 | anti-Dlar vs RbIgG LFQ | CG2233 |
| ***191*** | ***21.93557*** | ***16.68377*** | ***Glt-Fc vs Fc*** | ***Cg25C*** |
| ***38*** | ***24.1047*** | ***20.45928*** | ***Glt-Fc vs Fc LFQ*** | ***Cg25C*** |
| 187 | 22.72542 | 17.49358 | DlarFN45 vs RbIgG | CG32302 |
| 37 | 22.42609 | 18.80447 | DlarFN45 vs RbIgG LFQ | CG32302 |
| 21 | 23.51485 | 20.4807 | sDlar vs RbIgG | CG32521-RB;CG32521-RC |
| 62 | 25.3435 | 21.21833 | DlarFN45 vs RbIgG | CG4115 |
| 74 | 25.06595 | 20.76782 | DlarFN45 vs RbIgG LFQ | CG4115 |
| 17 | 24.76309 | 21.9512 | sDlar vs RbIgG | CG42486-RA |
| 36 | 21.88606 | 18.31079 | sDlar vs RbIgG | CG5080 |
| 16 | 20.26385 | 17.47684 | DlarIg12 vs RbIgG | CG6933-RA;CG6933-RC |
| 22 | 20.30575 | 17.19364 | sDlar-Fc vs Fc | CG7300 |
| 24 | 21.12288 | 17.94264 | sDlar vs RbIgG | CG7953 |
| 31 | 21.33342 | 17.9096 | sDlar vs RbIgG | CG8563-RA |
| 45 | 22.63823 | 18.82978 | sDlar vs RbIgG | CG9572-RB;CG9572-RA |
| 29 | 21.72205 | 18.34757 | sDlar vs RbIgG | Cht5 |
| 14 | 20.99438 | 18.34757 | anti-Dlar vs RbIgG | Cht5 |
| 12 | 27.86331 | 25.37262 | sDlar vs RbIgG | Clect27 |
| 36 | 26.78407 | 23.21021 | DlarFN45 vs RbIgG | Clect27 |
| 17 | 26.19935 | 23.38929 | DlarFN45 vs RbIgG LFQ | Clect27 |
| 13 | 20.05828 | 17.46348 | anti-Dlar(Ig12) vs RbIgG a | Cpr64Ad |
| 18 | 26.68016 | 23.80902 | sDlar vs RbIgG | CtsB1 |
| **11** | **21.12912** | **18.69379** | **DlarIg12 vs RbIgG LFQ** | **Dally** |
| ***294,537*** | ***33.28099*** | ***20.68782*** | ***Glt-Fc vs Fc*** | ***Glt*** |
| ***84,203,749*** | ***36.09203*** | ***17.84328*** | ***Glt-Fc vs Fc LFQ*** | ***Glt*** |
| 11 | 21.29709 | 18.88894 | sDlar vs RbIgG | Hml |
| 27 | 22.19874 | 18.88894 | anti-Dlar vs RbIgG | Hml |
| 81 | 21.57778 | 17.17867 | DlarIg12 vs RbIgG | Jon25Bi |
| 12 | 21.48075 | 19.01214 | DlarIg12 vs RbIgG LFQ | Jon25Bi |
| 154 | 22.2149 | 17.17867 | DlarFN45 vs RbIgG | Jon25Bi |
| 15 | 21.71079 | 19.01214 | DlarFN45 vs RbIgG LFQ | Jon25Bi |
| 15 | 20.78237 | 18.05059 | DlarIg12 vs RbIgG | Jon25Bii |
| 16 | 20.96564 | 18.20141 | DlarIg12 vs RbIgG LFQ | Jon25Bii |
| 72 | 22.32674 | 18.05059 | DlarFN45 vs RbIgG | Jon25Bii |
| 50 | 22.10348 | 18.20141 | DlarFN45 vs RbIgG LFQ | Jon25Bii |
| 84 | 26.37494 | 21.94646 | sDlar vs RbIgG | Jon25Biii |
| 39 | 25.03456 | 21.37836 | DlarIg12 vs RbIgG | Jon25Biii |
| 44 | 24.85253 | 21.07326 | DlarIg12 vs RbIgG LFQ | Jon25Biii |
| 48 | 25.24198 | 21.37836 | DlarFN45 vs RbIgG | Jon25Biii |
| 43 | 24.83904 | 21.07326 | DlarFN45 vs RbIgG LFQ | Jon25Biii |
| 28 | 21.87609 | 18.53061 | sDlar vs RbIgG | Jon65Ai |
| 23 | 21.80629 | 18.68608 | DlarFN45 vs RbIgG | Jon66Ci |
| 75 | 24.24133 | 19.92638 | DlarIg12 vs RbIgG | Jon99Ciii;Jon65Aii |
| 61 | 24.04143 | 19.92713 | DlarIg12 vs RbIgG LFQ | Jon99Ciii;Jon65Aii |
| 99 | 24.51875 | 19.92638 | DlarFN45 vs RbIgG | Jon99Ciii;Jon65Aii |
| 48 | 23.80449 | 19.92713 | DlarFN45 vs RbIgG LFQ | Jon99Ciii;Jon65Aii |
| 42 | 21.5871 | 17.85711 | sDlar vs RbIgG | Jon99Fii;Jon99Fi |
| 34 | 21.39263 | 17.85711 | anti-Dlar vs RbIgG | Jon99Fii;Jon99Fi |
| 79 | 21.904 | 17.53839 | DlarIg12 vs RbIgG | Jon99Fii;Jon99Fi;Jon44E |
| 14 | 21.857 | 19.19548 | DlarIg12 vs RbIgG LFQ | Jon99Fii;Jon99Fi;Jon44E |
| 67 | 21.74743 | 17.53839 | DlarFN45 vs RbIgG | Jon99Fii;Jon99Fi;Jon44E |
| **3,803,606** | **37.27396** | **22.12249** | **sDlar vs RbIgG LFQ** | **Lar** |
| **67,091** | **32.01704** | **20.90323** | **anti-Dlar vs RbIgG** | **Lar** |
| **21,871** | **32.11539** | **22.12249** | **anti-Dlar vs RbIgG LFQ** | **Lar** |
| **15,509** | **26.5796** | **16.9304** | **anti-Dlar(Ig12) vs RbIgG** | **Lar** |
| **805** | **25.30069** | **18.61043** | **anti-Dlar(Ig12) vs RbIgG LFQ** | **Lar** |
| **25,693** | **38.18935** | **28.03538** | **DlarFN46 vs CNTN4FN13** | **Lar** |
| **351,509** | **39.91277** | **27.14277** | **DlarFN46 vs CNTN4FN13 LFQ** | **Lar** |
| **4,030,657** | **35.01633** | **19.80689** | **sDlar-Fc vs Fc** | **Lar** |
| **1,612,329** | **35.54472** | **21.25153** | **sDlar-Fc vs Fc LFQ** | **Lar** |
| ***12,513*** | ***29.24141*** | ***19.80689*** | ***Glt-Fc vs Fc*** | ***Lar*** |
| ***8,533*** | ***30.30324*** | ***21.25153*** | ***Glt-Fc vs Fc LFQ*** | ***Lar*** |
| **10,472** | **27.35624** | **18.0998** | **anti-Dlar(Ig12) vs RbIgG a** | **Lar** |
| **3,389** | **25.44746** | **17.31921** | **anti-Dlar(Ig12) vs RbIgG LFQ a** | **Lar** |
| **462,069** | **31.14327** | **18.0998** | **DlarIg12 vs RbIgG** | **Lar** |
| **55,112** | **28.5229** | **17.60577** | **DlarIg12 vs RbIgG LFQ** | **Lar** |
| **586,695** | **31.38206** | **18.0998** | **DlarFN45 vs RbIgG** | **Lar** |
| **1,582,110** | **31.88004** | **17.60577** | **DlarFN45 vs RbIgG LFQ** | **Lar** |
| 11 | 20.78485 | 18.39534 | sDlar vs RbIgG | Loh |
| 13 | 23.81763 | 21.27333 | DlarIg12 vs RbIgG | Lsp1alpha |
| 13 | 23.70957 | 21.11 | DlarIg12 vs RbIgG LFQ | Lsp1alpha |
| 30 | 24.66221 | 21.27333 | DlarFN45 vs RbIgG | Lsp1alpha |
| 22 | 24.19746 | 21.11 | DlarFN45 vs RbIgG LFQ | Lsp1alpha |
| 14 | 30.30062 | 27.68224 | sDlar vs RbIgG | Lsp1gamma |
| 13 | 21.53274 | 18.95229 | DlarIg12 vs RbIgG | Muc55B |
| 18 | 21.53787 | 18.66364 | DlarIg12 vs RbIgG LFQ | Muc55B |
| 31 | 21.43781 | 18.0193 | sDlar vs RbIgG | Muc68Ca |
| 11 | 20.74222 | 18.32239 | DlarIg12 vs RbIgG LFQ | NimC1 |
| 13 | 20.8637 | 18.32239 | DlarFN45 vs RbIgG LFQ | NimC1 |
| 58 | 27.8649 | 23.79759 | sDlar vs RbIgG | Nw |
| 37 | 28.7631 | 25.14802 | sDlar vs RbIgG LFQ | Nw |
| 20 | 28.13531 | 25.14802 | anti-Dlar vs RbIgG LFQ | Nw |
| 12 | 20.47853 | 18.03159 | DlarIg12 vs RbIgG | PGRP-SB1 |
| 20 | 21.04634 | 18.03159 | DlarFN45 vs RbIgG | PGRP-SB1 |
| 83 | 25.91666 | 21.49882 | sDlar vs RbIgG | Ppn |
| 22 | 26.46145 | 23.38824 | sDlar vs RbIgG LFQ | Ppn |
| 25 | 24.70978 | 21.49882 | anti-Dlar vs RbIgG | Ppn |
| 18 | 26.26465 | 23.38824 | anti-Dlar vs RbIgG LFQ | Ppn |
| 104 | 23.37915 | 18.73437 | DlarIg12 vs RbIgG | Ppn |
| 89 | 23.15657 | 18.66605 | DlarIg12 vs RbIgG LFQ | Ppn |
| 353 | 24.59963 | 18.73437 | DlarFN45 vs RbIgG | Ppn |
| 315 | 24.41931 | 18.66605 | DlarFN45 vs RbIgG LFQ | Ppn |
| **43** | **22.03869** | **18.28534** | **DlarIg12 vs RbIgG LFQ** | **Sdc** |
| 141 | 22.42936 | 17.48385 | DlarIg12 vs RbIgG | sp151 |
| 84 | 22.38459 | 17.95657 | DlarIg12 vs RbIgG LFQ | sp151 |
| 162 | 22.57247 | 17.48385 | DlarFN45 vs RbIgG | sp151 |
| 69 | 22.18483 | 17.95657 | DlarFN45 vs RbIgG LFQ | sp151 |
| **289** | **24.80302** | **19.13796** | **DlarIg12 vs RbIgG** | **Swim** |
| **1,263** | **24.84953** | **17.70813** | **DlarIg12 vs RbIgG LFQ** | **Swim** |
| **29** | **22.07463** | **18.70361** | **DlarIg12 vs RbIgG LFQ** | **Trol** |
| 11 | 20.10482 | 17.70745 | DlarFN45 vs RbIgG | Vago |
| ***81*** | ***20.78732*** | ***16.39874*** | ***Glt-Fc vs Fc*** | ***Vkg*** |

**^1^ Student's T-test significance, p<0.05**

**^2^ Dlar and associated proteins of interest**

**^3^ *Glt and associated* *proteins of interest***
