# Supplementary material for: Complex protein interactions mediate Drosophila Lar function in muscle tissue
Source: PLoS One. 2022 May 27;17(5):e0269037. doi: 10.1371/journal.pone.0269037 (PMC9140312; doi:10.1371/journal.pone.0269037)
Supplement: S4 Table — (DOCX) [file pone.0269037.s004.docx]

**S4 Table. Proteins identified in LC-MS/MS that co-purify with Mammalian PTPRD or LAR baits.**

| **Enrichment^1^** | **Sample intensity (log2)** | **Control Intensity (Log2)** | **Bait vs. Control** | **Protein^2^** | **Protein - Cell line** | ***Drosophila* homolog** |
| --- | --- | --- | --- | --- | --- | --- |
| 59 | 27.99016 | 23.91841 | PTPRD_CNTN1+6 | A1m | PTPRD - C6 | Tep2, Tep4? |
| 1,261 | 30.84461 | 23.7051 | PTPRD_CNTN1+6 | Adgrg1 | PTPRD - B35 | CG11318, Cirl? |
| 617 | 29.19213 | 22.76675 | PTPRD_CNTN1+6 | Angptl2 | PTPRD - C2C12 | CG41520, 30281, 30280 |
| 75,160 | 33.38998 | 22.16261 | PTPRD_CNTN1+6 | Apod | PTPRD - B35 | Nlaz |
| 13 | 25.77442 | 23.20596 | PTPRD_CNTN1+6 | Bsg | PTPRD - C6 | Bsg |
| 18 | 29.24459 | 26.37393 | LAR_CNTN1+6 | Bsg | LAR - C6 | Bsg |
| 821 | 28.29807 | 21.58786 | PTPRD_CNTN1+6 | C3 | PTPRD - C2C12 | Tep2, Tep1, Tep4? |
| 164 | 27.65492 | 22.5549 | LAR_CNTN1+6 | Cd99 | LAR - C6 | no significant hit |
| 47 | 29.88983 | 26.04015 | PTPRD_CNTN1+6 | Chrdl1 | PTPRD - C6 | Sog |
| **50** | **22.87581** | **18.97136** | **LAR_CNTN1+6** | **Col1a1** | **LAR - C2C12** | **Vkg/Col4a1** |
| 13 | 24.89712 | 22.35587 | PTPRD_CNTN1+6 | Ctsb | PTPRD - C6 | Ctsb1 |
| 18 | 27.36611 | 24.4576 | LAR_CNTN1+6 | Ctsb | LAR - C6 | Ctsb1 |
| 32 | 25.97482 | 22.49776 | PTPRD_CNTN1+6 | Ctsd | PTPRD - C6 | CathD |
| 25,506 | 31.89558 | 21.74891 | PTPRD_CNTN1+6 | Ctsf | PTPRD - B35 | CG12163 |
| 26 | 25.35642 | 22.08314 | LAR_CNTN1+6 | Ctsf | LAR - C6 | CG12163 |
| **36** | **22.77983** | **19.18565** | **PTPRD_CNTN1+6** | **Dag1** | **PTPRD - C6** | **Dg** |
| **241** | **27.68432** | **22.20015** | **LAR_CNTN1+6** | **Dag1** | **LAR - C6** | **Dg** |
| 96,839 | 34.46069 | 22.97988 | PTPRD_CNTN1+6 | Dhh | PTPRD - B35 | Hh |
| 10 | 27.90518 | 25.55421 | PTPRD_CNTN1+6 | Emb | PTPRD - Neuro2a | Dip-eta |
| 13 | 25.01954 | 22.44803 | LAR_CNTN1+6 | Emb | LAR - Neuro2a | Dip-eta |
| 31 | 29.73688 | 26.30634 | LAR_CNTN1+6 | Grn | LAR - C6 | no significant hit |
| **494** | **28.59249** | **22.3892** | **PTPRD_CNTN1+6** | **Hspg2** | **PTPRD - C2C12** | **Trol** |
| 309 | 24.87061 | 19.13603 | LAR_CNTN1+6 | Hspg2 | LAR - C2C12 | Trol |
| 13 | 24.52546 | 21.92719 | LAR_CNTN1+6 | Hspg2 | LAR - Neuro2a | Trol |
| 18 | 25.27803 | 22.38131 | PTPRD_CNTN1+6 | Igf2r | PTPRD - C6 | Lerp |
| 33 | 25.57039 | 22.06979 | PTPRD_CNTN1+6 | Il1rap | PTPRD - C6 | Tollo |
| **12** | **33.09388** | **30.5823** | **PTPRD_CNTN1+6** | **Itga1** | **PTPRD - C6** | **αPS1 integrin/mew** |
| **17** | **25.32334** | **22.50592** | **PTPRD_CNTN1+6** | **Itga5** | **PTPRD - C6** | **αPS1 integrin/if** |
| **42** | **23.174** | **19.43974** | **PTPRD_CNTN1+6** | **Itga6** | **PTPRD - C6** | **αPS1 integrin/mew** |
| **18** | **26.85043** | **23.95673** | **LAR_CNTN1+6** | **Itga6** | **LAR - C6** | **αPS1 integrin/mew** |
| **24** | **25.85816** | **22.69468** | **PTPRD_CNTN1+6** | **Itga7** | **PTPRD - C6** | **αPS1 integrin/mew** |
| **14** | **28.54004** | **25.91737** | **PTPRD_CNTN1+6** | **Itgb1** | **PTPRD - C6** | **βPS1 integrin/mys** |
| **11** | **27.91974** | **25.49579** | **LAR_CNTN1+6** | **Itgb1** | **LAR - C6** | **βPS1 integrin/mys** |
| 111 | 27.82469 | 23.11092 | PTPRD_CNTN1+6 | Itih1 | PTPRD - C6 | no significant hit |
| 1,119 | 28.59783 | 21.57727 | LAR_CNTN1+6 | Itih1 | LAR - C6 | no significant hit |
| 16 | 24.82886 | 22.08617 | PTPRD_CNTN1+6 | Lgals1 | PTPRD - C6 | Galectin |
| 13 | 33.10878 | 30.5823 | LAR_CNTN1+6 | Lgals1 | LAR - C6 | Galectin |
| 28 | 25.53668 | 22.21152 | LAR_CNTN1+6 | Lgmn | LAR - C6 | PIG-K |
| 19 | 29.24923 | 26.31835 | LAR_CNTN1+6 | Lrpap1 | LAR - C6 | CG8507 |
| 49 | 26.92298 | 23.03936 | PTPRD_CNTN1+6 | Ltbp3 | PTPRD - C6 | frac, dpy |
| 145 | 27.36595 | 22.39017 | LAR_CNTN1+6 | Ltbp3 | LAR - C6 | frac, dpy |
| 17 | 25.3963 | 22.56957 | PTPRD_CNTN1+6 | M6pr | PTPRD - C6 | no significant hit |
| 98 | 26.83846 | 22.24983 | PTPRD_CNTN1+6 | Mfge8 | PTPRD - C6 | Hml |
| 306 | 27.89954 | 22.17464 | LAR_CNTN1+6 | Mfge8 | LAR - C6 | Hml |
| 17 | 25.08257 | 22.23932 | LAR_CNTN1+6 | Mgp | LAR - C6 | no significant hit |
| 12 | 24.98802 | 22.46601 | PTPRD_CNTN1+6 | Mlec | PTPRD - C6 | CG9257 |
| 14 | 32.98019 | 30.31883 | LAR_CNTN1+6 | Mlec | LAR - C6 | CG9257 |
| 63 | 28.1032 | 23.95673 | PTPRD_CNTN1+6 | Mrc2 | PTPRD - C6 | no significant hit |
| 161 | 27.52753 | 22.446 | LAR_CNTN1+6 | Mrc2 | LAR - C6 | no significant hit? |
| 425 | 27.62742 | 21.57571 | PTPRD_CNTN1+6 | Mxra7 | PTPRD - C2C12 | CG7407? |
| 3,090 | 27.1048 | 19.06883 | PTPRD_CNTN1+6 | Oaf | PTPRD - B35 | Oaf |
| 48 | 26.73518 | 22.85765 | PTPRD_CNTN1+6 | Oaf | PTPRD - C6 | Paf |
| 47 | 26.72806 | 22.87926 | PTPRD_CNTN1+6 | Plxnb2 | PTPRD - C6 | PlexA, PlexB |
| 49 | 26.55009 | 22.66676 | LAR_CNTN1+6 | Plxnb2 | LAR - C6 | PlexA, PlexB |
| 11 | 32.6785 | 30.31883 | PTPRD_CNTN1+6 | Psap | PTPRD - Neuro2a | Sap-r |
| 11 | 28.9749 | 26.56358 | LAR_CNTN1+6 | Psap | LAR - C6 | Sap-r |
| **784,844** | **35.27284** | **21.6996** | **PTPRD_CNTN1+6** | **Ptprd** | **PTPRD - B35** | **Dlar** |
| **839** | **29.19387** | **22.46202** | **PTPRD_CNTN1+6** | **Ptprd** | **PTPRD - C2C12** | **Dlar** |
| **282** | **27.86591** | **22.2226** | **PTPRD_CNTN1+6** | **Ptprd** | **PTPRD - C6** | **Dlar** |
| **12** | **27.9741** | **25.49579** | **PTPRD_CNTN1+6** | **PTPRD** | **PTPRD - HEK293** | **Dlar** |
| **11** | **26.88309** | **24.4576** | **PTPRD_CNTN1+6** | **Ptprd** | **PTPRD - Neuro2a** | **Dlar** |
| **59** | **26.6569** | **22.58327** | **LAR_CNTN1+6** | **Ptprd** | **LAR - C6** | **Dlar** |
| **194,025** | **34.306** | **22.13026** | **LAR_CNTN1+6** | **Ptprf** | **LAR - B35** | **Dlar** |
| **102,498** | **33.6345** | **22.0969** | **PTPRD_CNTN1+6** | **Ptprf** | **PTPRD - B35** | **Dlar** |
| **623** | **28.17455** | **21.73938** | **PTPRD_CNTN1+6** | **Ptprf** | **PTPRD - C2C12** | **Dlar** |
| **245** | **27.87929** | **22.37801** | **PTPRD_CNTN1+6** | **Ptprf** | **PTPRD - C6** | **Dlar** |
| **12** | **28.82854** | **26.37393** | **PTPRD_CNTN1+6** | **PTPRF** | **PTPRD - HEK293** | **Dlar** |
| **11** | **21.59905** | **19.19614** | **PTPRD_CNTN1+6** | **Ptprf** | **PTPRD - Neuro2a** | **Dlar** |
| **27,479** | **34.13958** | **23.91841** | **LAR_CNTN1+6** | **Ptprf** | **LAR - C2C12** | **Dlar** |
| **598,607** | **37.06527** | **23.76291** | **LAR_CNTN1+6** | **Ptprf** | **LAR - C6** | **Dlar** |
| **23,097** | **31.62318** | **21.57571** | **LAR_CNTN1+6** | **PTPRF** | **LAR - HEK293** | **Dlar** |
| **148,840** | **33.87482** | **21.96419** | **LAR_CNTN1+6** | **Ptprf** | **LAR - Neuro2a** | **Dlar** |
| 24 | 25.69123 | 22.52002 | PTPRD_CNTN1+6 | Serpinc1 | PTPRD - C6 | Spn42Da |
| 29 | 25.1102 | 21.7578 | LAR_CNTN1+6 | Slc1a4 | LAR - C6 | Eaat1, Eaat2 |
| 37 | 25.78014 | 22.15934 | LAR_CNTN1+6 | Slc1a5 | LAR - C6 | Eaat1, Eaat2 |
| 11 | 26.61246 | 24.19382 | LAR_CNTN1+6 | Vps37b | LAR - C6 | Vps37b |
| 3,426 | 31.41986 | 23.28057 | PTPRD_CNTN1+6 | Vwa1 | PTPRD - B35 | no significant hit |

**^1^ Student's T-test significance, p<0.05**

**^2^ LAR/RTPRD and associated proteins of interest**
