# Supplementary figures and images for: Complex protein interactions mediate Drosophila Lar function in muscle tissue
Source: PLoS One. 2022 May 27;17(5):e0269037. doi: 10.1371/journal.pone.0269037 (PMC9140312; doi:10.1371/journal.pone.0269037)

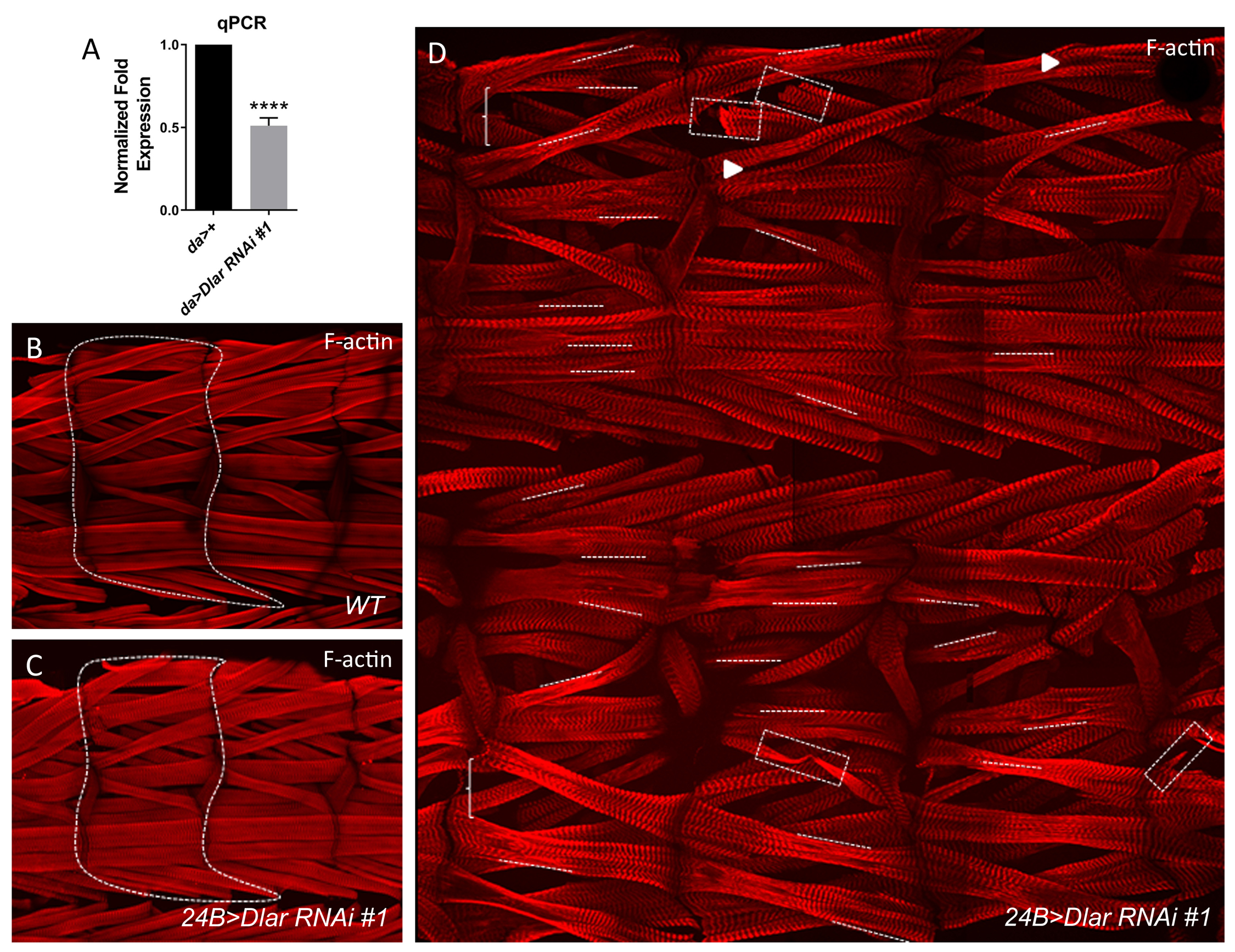

Supplement: S1 Fig — (A) Bar graph depicting relative dlar transcript levels in control (da>+) or knockdown (da>Dlar RNAi #1) larvae. Mean +/- SD. ****, p< 0.001. N = 3 biological replicates and 3 technical replicates for each genotype. (B-D) Immunofluorescence of F-actin in L3 muscle. Left is anterior in all images. (B) Two complete hemisegments of WT musculature. White dashed line outlines a single hemisegment. (C) Overall muscle patterning is normal in 24B>Dlar RNAi #1 larvae. White dashed line outlines a single hemisegment. (D) Composite image of six hemisegments of 24B>Dlar RNAi #1 muscles. Sarcomeric patterning defects (white dashed line), splits in myofibrils (arrowhead), broken or tearing muscle (boxes), increase in distance between adjacent dorsal myofibers (solid braces) can be observed. (TIF) [file pone.0269037.s005.tif]

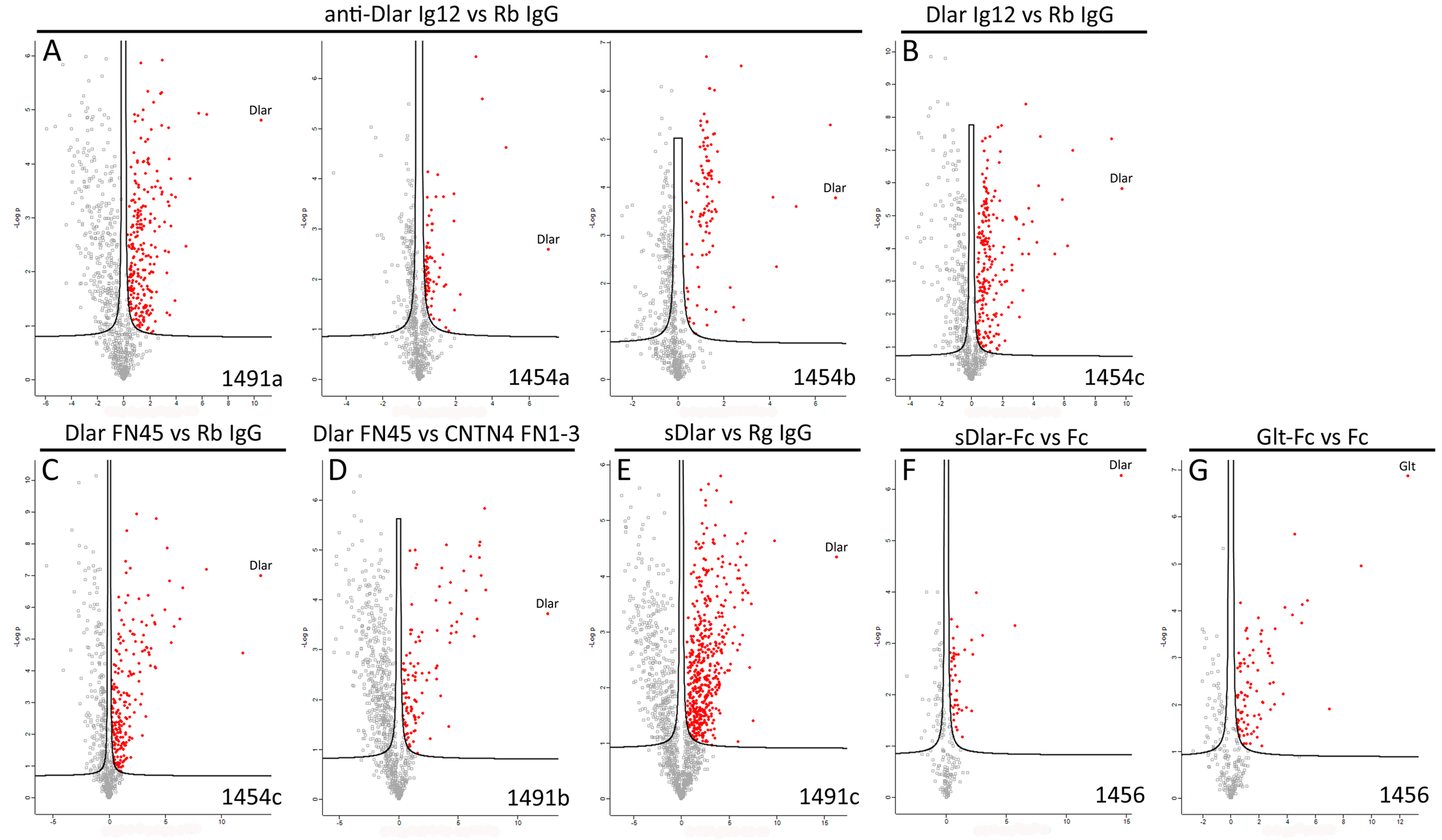

Supplement: S2 Fig — (A-G) Volcano scatterplots showing the relative number of proteins identified in individual Dlar pulldown experiments. Y-axis shows statistical significance (p<o.o5) and x-axis depicts fold change. (TIF) [file pone.0269037.s006.tif]

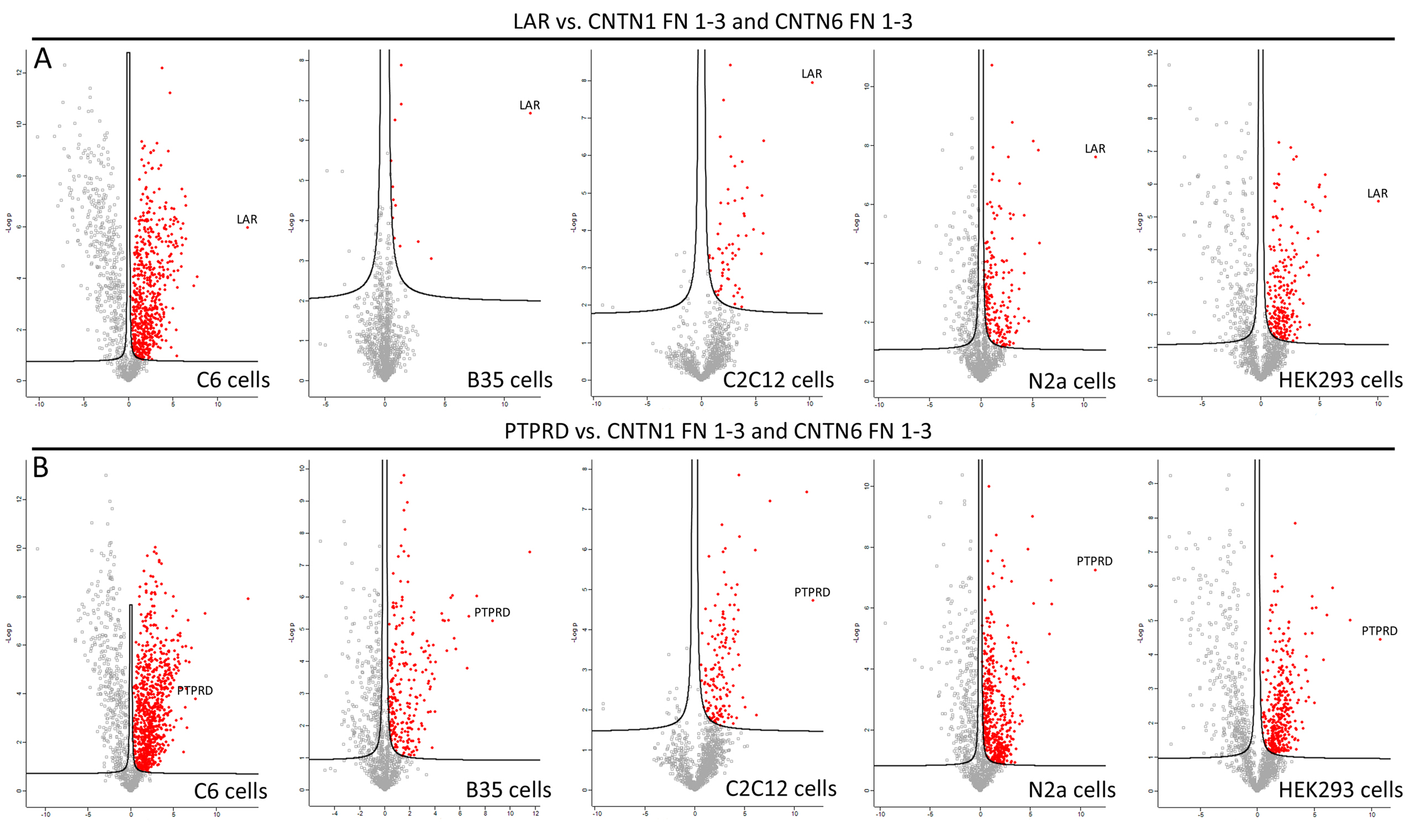

Supplement: S3 Fig — (A,B) Volcano scatterplots showing the relative number of proteins identified in individual pulldown experiments in the indicated cell types. Y-axis shows statistical significance (p<o.o5) and x-axis depicts fold change. (TIF) [file pone.0269037.s007.tif]

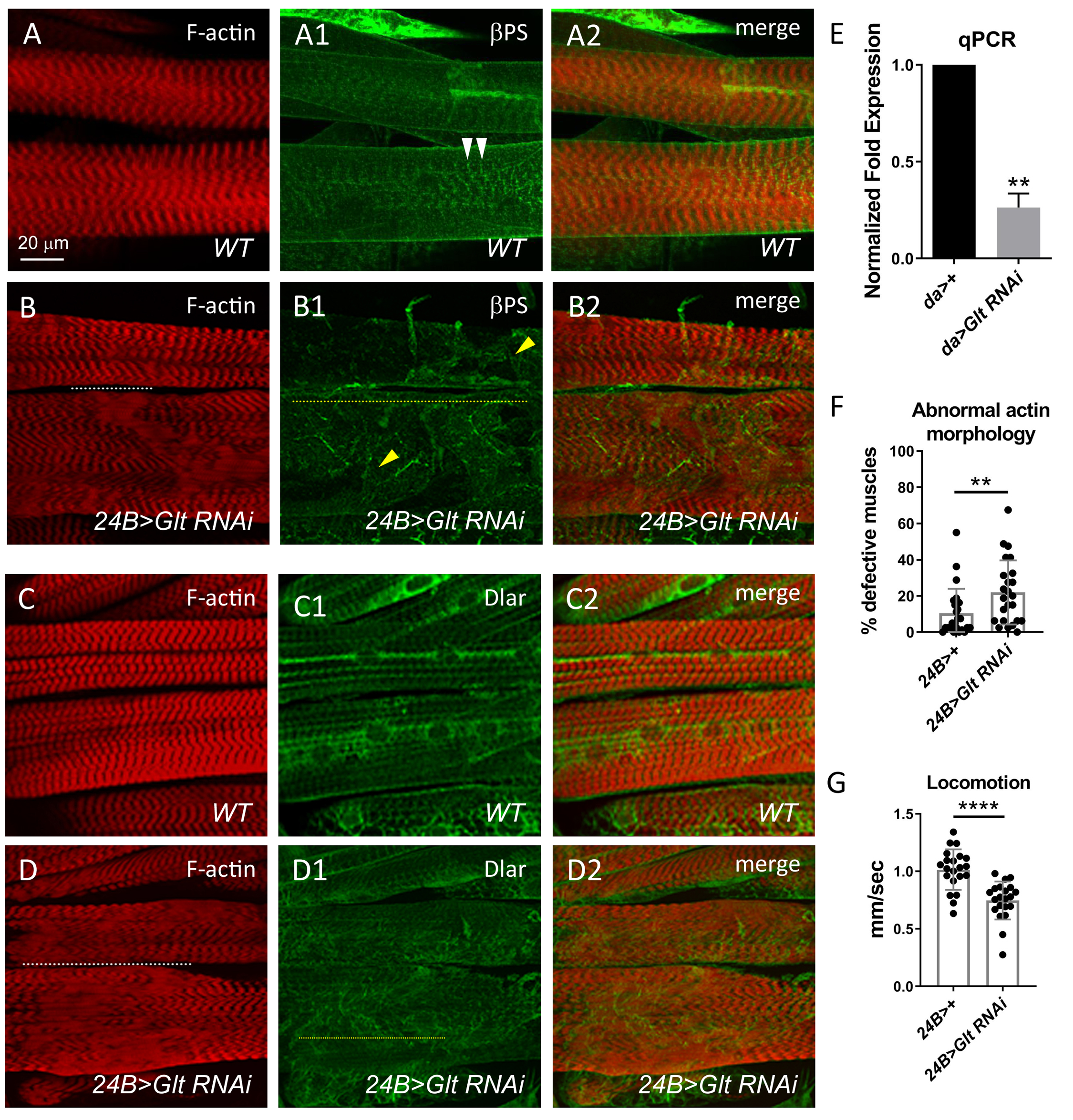

Supplement: S4 Fig — (A-D2) Immunofluorescence of βPS integrin (A-B2, green) or Dlar (C-D2, green) in WT (A-A2,C-C2) or 24B>Glt RNAi (B-B2,D-D2) co-stained with phalloidin (red) in L3 larval muscle. White arrowheads indicate βPS striations. Yellow arrowheads show regions of torn or damaged sarcolemma. Yellow dotted line highlight regions of abnormal patterning. (E) Bar graph depicting relative Glt transcript levels in control (da>+) or knockdown (da>Glt RNAi) larvae. Mean +/- SD. **, p< 0.01. N = 3 biological replicates and 3 technical replicates for each genotype. (F,G) Scatter and bar graphs representing abnormal muscle defects assayed by F-actin patterning (F) or locomotor ability of L3 larvae (G) in WT or 24B>Glt RNAi. Mean +/- SD. ****, p<0.001; **, p< 0.01. N ≥ 20. (TIF) [file pone.0269037.s008.tif]

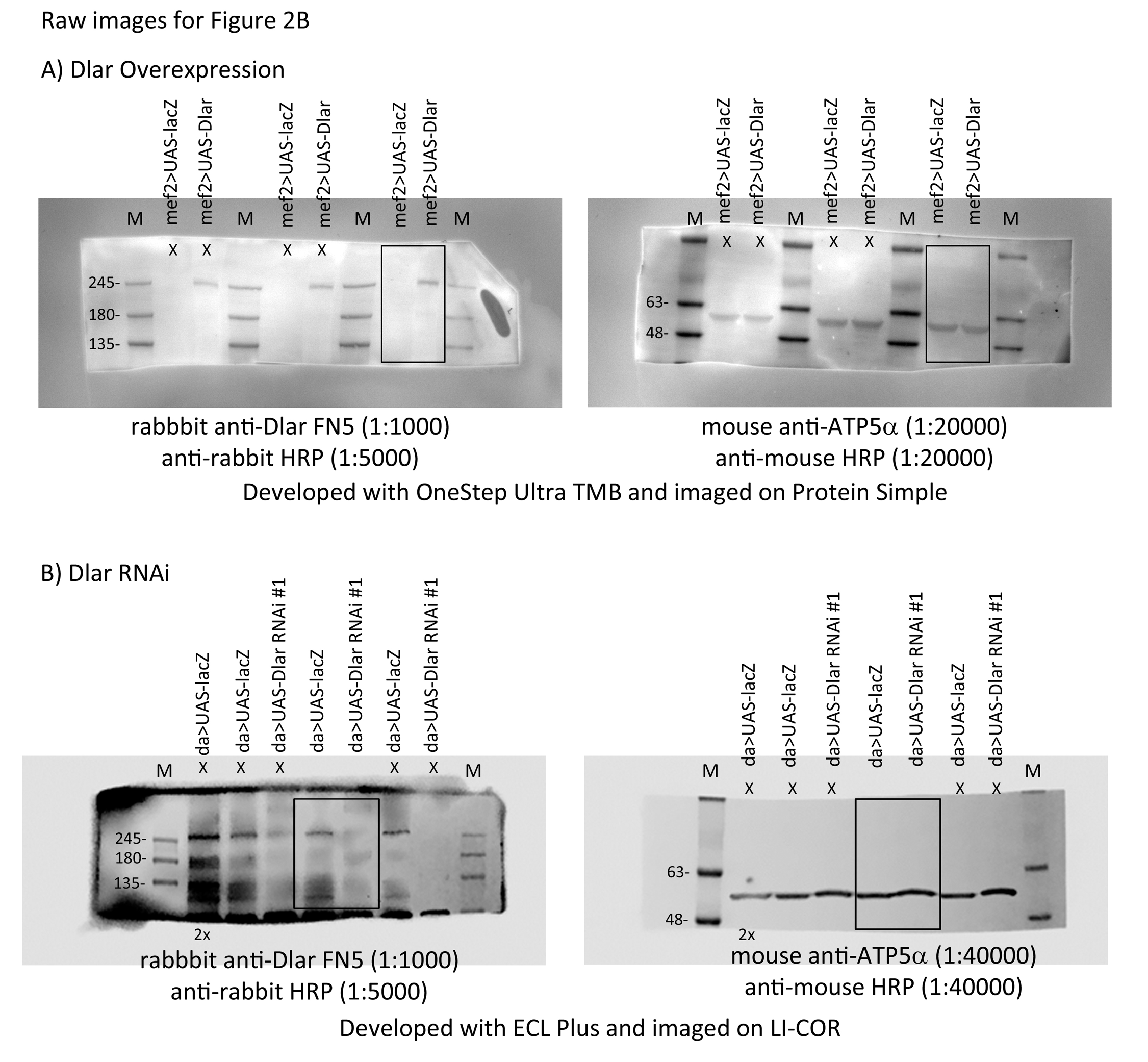

Supplement: S1 Raw images — Original, uncropped scans for Western blots in Fig 2B. Boxed rectangle in each blot corresponds to the lanes of cropped regions in Fig 2B, while the ‘X’ indicates biological replicates. Whole larvae were homogenized in SDS sample buffer, boiled 10 minutes, centrifuged to remove debris, and run on a 7% Tris-Glycine SDS-PAGE gel. (A) UAS-based overexpression of Dlar in muscle tissue using mef2-Gal4. (B) Knockdown of Dlar RNAi in all tissues using da-Gal4. ‘2x’ under blot indicates that twice the amount of da>lacZ lysate was loaded into this lane compared to other da>lacZ control lanes. (TIF) [file pone.0269037.s009.tif]
